# Mechanochemical Synthesis and Molecular Docking Studies of New Azines Bearing Indole as Anticancer Agents

**DOI:** 10.3390/molecules28093869

**Published:** 2023-05-04

**Authors:** Mohamed S. Ibrahim, Basant Farag, Jehan Y. Al-Humaidi, Magdi E. A. Zaki, Maher Fathalla, Sobhi M. Gomha

**Affiliations:** 1 Department of Chemistry, Faculty of Science, Islamic University of Madinah, Madinah 42351, Saudi Arabia; 431001336@stu.iu.edu.sa (M.S.I.); maherfathalla@iu.edu.sa (M.F.); 2 Department of Chemistry, Faculty of Science, Zagazig University, Zagazig 44519, Egypt; basantfarag@zu.edu.eg; 3 Department of Chemistry, College of Science, Princess Nourah bint Abdulrahman University, P.O. Box 84428, Riyadh 11671, Saudi Arabia; jyalhamidi@pnu.edu.sa; 4 Department of Chemistry, Faculty of Science, Imam Mohammad Ibn Saud Islamic University (IMSIU), Riyadh 11623, Saudi Arabia; mezaki@imamu.edu.sa; 5 Department of Chemistry, Faculty of Science, Cairo University, Cairo 12613, Egypt

**Keywords:** indole, azines, condensation, grinding, molecular docking, CDK-5 and SAR

## Abstract

The development of new approaches for the synthesis of new bioactive heterocyclic derivatives is of the utmost importance for pharmaceutical industry. In this regard, the present study reports the green synthesis of new benzaldazine and ketazine derivatives via the condensation of various carbonyl compounds (aldehydes and ketones with the 3-(1-hydrazineylideneethyl)-1*H*-indole using the grinding method with one drop of acetic acid). Various spectroscopic techniques were used to identify the structures of the synthesized derivatives. Furthermore, the anticancer activities of the reported azine derivatives were evaluated against colon, hepatocellular, and breast carcinoma cell lines using the MTT technique with doxorubicin as a reference medication. The findings suggested that the synthesized derivatives exhibited potential anti-tumor activities toward different cell lines. For example, **3c**, **3d**, **3h**, **9**, and **13** exhibited interesting activity with an IC_50_ value of 4.27–8.15 µM towards the HCT-116 cell line as compared to doxorubicin (IC_50_ = 5.23 ± 0.29 µM). In addition, 3c, 3d, 3h, 9, 11, and 13 showed excellent cytotoxic activities (IC_50_ = 4.09–9.05 µM) towards the HePG-2 cell line compared to doxorubicin (IC_50_ = 4.50 ± 0.20 µM), and 3d, 3h, 9, and 13 demonstrated high potency (IC_50_ = 6.19–8.39 µM) towards the breast cell line (MCF-7) as compared to the reference drug (IC_50_ = 4.17 ± 0.20 µM). The molecular interactions between derivatives **3a-h**, **7**, **9**, **11**, **13**, and the CDK-5 enzyme (PDB ID: 3IG7) were studied further using molecular docking indicating a high level of support for the experimental results. Furthermore, the drug-likeness analysis of the reported derivatives indicated that derivative **9** (binding affinity = −8.34 kcal/mol) would have a better pharmacokinetics, drug-likeness, and oral bioavailability as compared to doxorubicin (−7.04 kcal/mol). These results along with the structure–activity relationship (SAR) of the reported derivatives will pave the way for the design of additional azines bearing indole with potential anticancer activities.

## 1. Introduction

Cancer is the second most common cause of death after heart disease. Cancer is the uncontrolled, rapid, and pathological proliferation of abnormal cells [1]. It claims the lives of over 9.6 million people each year [2]. Despite significant advances in the discovery of anticancer medications, there are still several barriers to cancer therapy, such as low effectiveness, excessive toxicity, and drug resistance, all of which have severely impacted patients’ normal lives [3]. As a result, one of the most important areas of current cancer research is the hunt for strong, safe anticancer agents with great selectivity [4]. Compounds possessing heterocyclic core play a significant role in designing and developing a new class of structural entities for pharmaceutical applications. Indoles represent an important structural class in drug discovery due to its important biological activities including antibacterial, antifungal [5,6], anti-inflammatory [7], antihistamine [8], antioxidant [9], anti-diabetic [10], antiviral [11], anticholinesterase [12] as well as anticancer properties, like vincristine, vinblastine, koumine, and topsentine [13,14,15,16,17,18,19,20].

On the other hand, aldazines and ketazines with a conjugated hydrazone system (C=N-N=C) were exploited in various pharmacological studies and many commercial applications. Both symmetric and asymmetric azines have demonstrated biological activities as antimicrobial, antibacterial, anti-inflammatory, antimalarial dyes, anticonvulsant, insect growth regulating, and many other activities [21,22,23,24,25]. Furthermore, several aldazine and ketazine derivatives possessed antiproliferative properties and were applied as cytotoxic agents capable of preventing cell progression in malignant cells via various mechanisms [26,27,28]. Consequently, indole hydrazones demonstrated selective and efficient cytotoxicity owing to their important favorable pharmacological properties, low toxicity, and low undesirable effects [29,30,31].

Green synthesis is an emerging field that offers significant economic and environmental advantages [32]. In this respect, choosing the reaction solvent, catalyst, and technique based on green chemistry protocols (GCPs) is of the utmost importance [33,34,35,36,37,38,39,40]. It is also vital to avoid the creation of unwanted or harmful byproducts by developing dependable, sustainable, and environmentally friendly synthesis processes [41,42,43]. The advantages of green chemistry protocols (GCPs) were harnessed by pharmaceutical production and implemented all (or most) of GCPs as far as practicable.

Recently, molecular docking was employed by many researchers as an effective tool to stimulate the potential interactions between two entities, thus guiding the design of effective therapeutic derivatives through the evaluation of its binding affinities with various enzymes [44,45,46]. In is reported that cyclin-dependent kinases (CDK) play key roles in the cell cycle, and CDK inhibitors have been widely investigated as cancer therapy agents [47], where vital interactions with residues of amino acids Asn132, Glu81, Glu12, Lys89, Gln85, Asp86, Lys129, Val18, Gln131, Lys33, Gly13, and Gly11 were demonstrated by molecular docking investigations. It was possible to determine the binding interactions of the produced derivatives to the EFP as native ligand [48,49].

Bearing the aforementioned in mind, and in the framework of our ongoing research on the synthesis of bioactive heterocyclic derivatives [42,50,51,52,53,54,55,56,57,58,59], the goal of the present study is the green synthesis of some new aldazine and ketazine derivatives, starting with 3-(1-hydrazineylideneethyl)-1*H*-indole and the evaluation of their potential anticancer activities, which was further supported by molecular docking studies using EFP as a native ligand [48,49]. In addition, the drug-likeness as well as SAR analysis of the reported derivatives were also investigated.

## 2. Results and Discussion

### 2.1. Chemistry

**Scheme 1:**
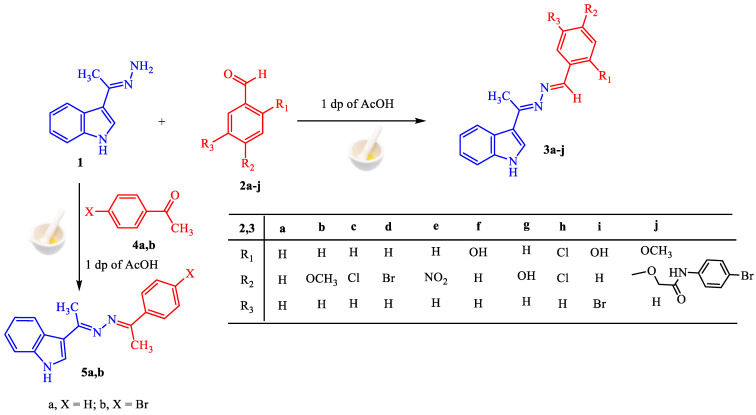
Synthesis of aldazines **3a**–**j** and ketazines **5a**,**b.**

The (E)-3-(1-hydrazineylideneethyl)-*1H*-indole **1** [60] is a critical precursor for the synthesis of the proposed derivatives, utilizing facile and green methodology. The condensation of carbonyl compounds (aldehydes and ketones) with hydrazine **1** afforded the corresponding azines under grinding process with one drop of acetic acid. For examples, the reaction of the hydrazine **1** with aromatic benzaldehyde derivatives **2a**–**j** give the corresponding benzaldazine derivatives **3a**–**j** in high to exceptional yields regardless of the kind of substituents (electron-withdrawing or electron-donating group) as shown in Scheme 1.

Based on information from their ^1^H-NMR, mass, IR, and elemental analyses, the structure of derivatives **3a**–**j** was validated. For the ^1^H-NMR spectra of the condensation products **3a**–**j**, all lack the signal attributed to NH_2_ groups indicating azine formation. In addition, the imine proton CH=N- is clearly apparent at *δ* ~ 8.68 ppm, as well as in the presence of the expected aromatic protons. In the IR spectra, the stretching absorption of the (C=N) group was observed at *v* ~ 1593 cm^−1^. Elemental analyses accord well with the hypothesized structures **3a**–**j**.

The efficacy of grinding as compared to the traditional heating was evaluated by comparing the yield and time of the reaction of the two methodologies. Table 1 showed that the application of the grinding process is preferable for the reported reactions, as it sped up the reaction time and increased product yields.

The ketazine series is extended by reaction of hydrazine **1,** with aromatic ketones **5a**,**b** using the grinding method to furnish ketazines **6a**,**b**, respectively. The synthesis is simple and efficient, since nothing is added except starting ingredients and one drop of AcOH impacted by mechanical power (Scheme 1). The structure of ketazine derivatives **6a**,**b** were confirmed using all available spectroscopic techniques (see experimental part).

**Scheme 2:**
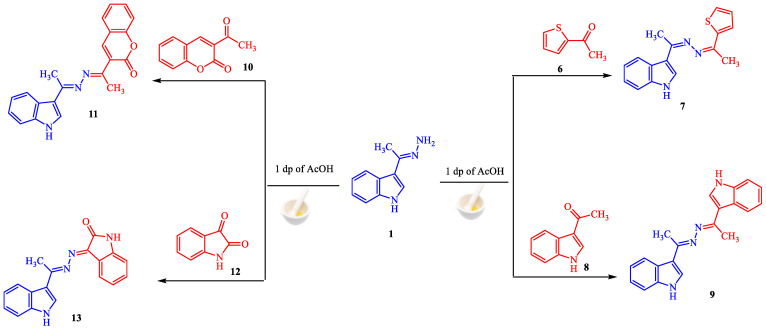
Synthesis of ketazines **7**, **9**, **11** and **13**.

Our efforts were extended to the preparation of symmetric and asymmetric heterocyclic ketazines. Thus, different acetyl heterocyclic compounds (**6**, **8**, and **10**) and isatin **12** were subjected to react with hydrazine **1** under the employed conditions to give the respective ketazines **7**, **9**, **11**, and **13** in good yields (Scheme 2). The structures of these compounds are validated by their spectroscopic data. For example, the ^1^H-NMR spectrum of azine **13** showed three singlet signals at *δ* 2.46, 9.54, and 10.64 ppm for methyl group and two NH groups. The nine aromatic protons were resonated as multiplet signals at *δ* 6.81–8.26 ppm. Moreover, its mass analysis identified the molecular ion peak at *m*/*z* = 302 (37%), This is equivalent to a chemical formula (C_18_H_14_N_4_O).

#### 2.1.1. Biological Evaluation

**Figure 1:**
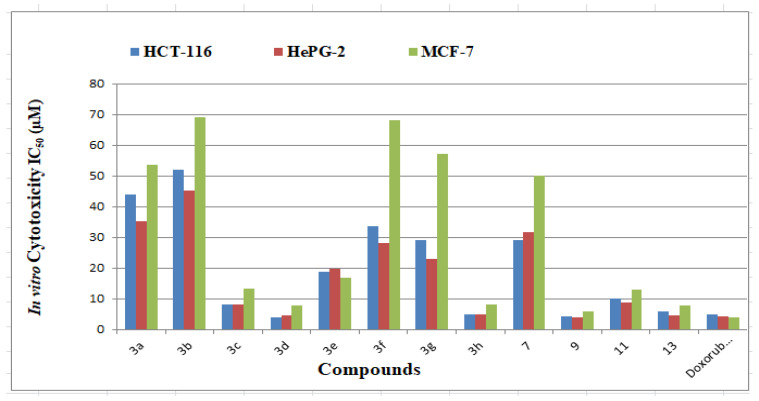
IC_50_ of the cytotoxic activity of the examined derivatives against human tumor cell lines.

The synthesized azine derivatives were tested in vitro for inhibitory efficacy versus anticancer cell lines like HCT-116 (colorectal carcinoma), HEPG-2 (hepatocellular carcinoma), and MCF-7 (human breast adenocarcinoma) using the MTT technique. Doxorubicin, a commonly used small-molecule anticancer medication, was used as a control. The concentration of chemicals required to inhibit the development of 50% of cancer cells was represented as (μM) and is shown in Table 2 and Figure 1.

The 12 derivatives under investigation inhibit human tumor cells to various degrees. When compared to doxorubicin (IC_50_ = 5.23 ± 0.29 µM), derivatives **3a**–**h**, **7**, **9**, **11**, and **13** were tested for their capacity to inhibit the HCT-116 cell line. The synthesized derivatives’ inhibitory potency ranged from 4.27 to 52.28 µM. Derivatives **3c**, **3d**, **3h**, **9**, and **13** exhibited interesting activity with an IC_50_ value of 4.27–8.15 µM. Further, compounds 3e, 3f, 3g, 7, and 11 are proved to be of moderate activity with IC_50_ values of 10.18–33.18 µM, respectively. On the other hand, derivatives 3a and 3b demonstrated poor activity with IC_50_ values >40 µM.

The 12 HCT-116 inhibitors obtained were selected to be further assayed against the HePG-2 cell line and compared with doxorubicin (IC_50_ = 4.50 ± 0.20 µM). Derivatives 3c, 3d, 3h, 9, 11, and 13 showed excellent cytotoxic activities (IC_50_ = 4.09–9.05 µM). Moderate cytotoxic activities were noticed by derivatives 3a, 3f, 3g and 7 with IC_50_ values = 19.78–31.71 µM. Moreover, derivatives 3a and 3b displayed low values of IC_50_ > 35 µM. Finally, many derivatives were evaluated against the breast cell line (MCF-7). Compounds 3d, 3h, 9, and 13 demonstrated high potency (IC_50_ = 6.19–8.39 µM) compared to the reference drug (IC_50_ = 4.17 ± 0.20 µM). Derivatives 3c, 3e, and 11 proved to be moderately active with IC_50_ = 13.27–17.02 µM. The other five azines are inactive and registered IC_50_ > 50 µM. Based on earlier research, we discovered that HePG-2 was the most effective growth inhibitor among the cells tested, while MCF-7 was the least effective.

#### 2.1.2. SAR Analysis for Aldazine Derivatives **3a**–**h**

Investigations into the data from the evaluation of the analyzed derivatives’ inhibitory effects against three different tumor cell lines make it clear that certain structural characteristics of the tested derivatives are necessary for specific activities to exist. Based on previous findings, it was discovered that substituting the phenyl ring with a mono substituent bromo atom at the para position in derivative **3d** and a di substituent chloro atom at the ortho and para positions in derivative **3h** resulted in the most potent cytotoxic activities vs. the reference medication, doxorubicin, against the HCT-116 cell line. Introduction of the Cl group was a small size electron-withdrawing moiety, which also included**3c** high activity, while nitro group **3e** is bulky and steric. In general, derivatives with *meta* substitution on the phenyl ring **3f** were less potent than those with *para*-substituted derivatives **3g** [61]. Finally, derivatives **3a** and **3b** had weak activity against three cell lines.

#### 2.1.3. SAR Analysis for Ketazine Derivatives **7**, **9**, **11** and **13**

Based on the past outcomes, it was noticed that two indole rings in derivative **9** gave the most potent cytotoxic activities against HePG-2 and HCT-116 cell lines in comparison with the reference drug, doxorubicin. In addition, the analogs with 5-membered aromatic heterocycle exhibited higher inhibitory activity than 6-membered aromatic heterocycle analogs [62,63]. Therefore, the derivative with indolin-2-one ring **13** displayed stronger inhibitory ability than compound with chromen-2-one ring **11**. Finally, derivative **7** had weak activity against three cell lines.

### 2.2. Molecular Docking Studies

**Figure 2:**
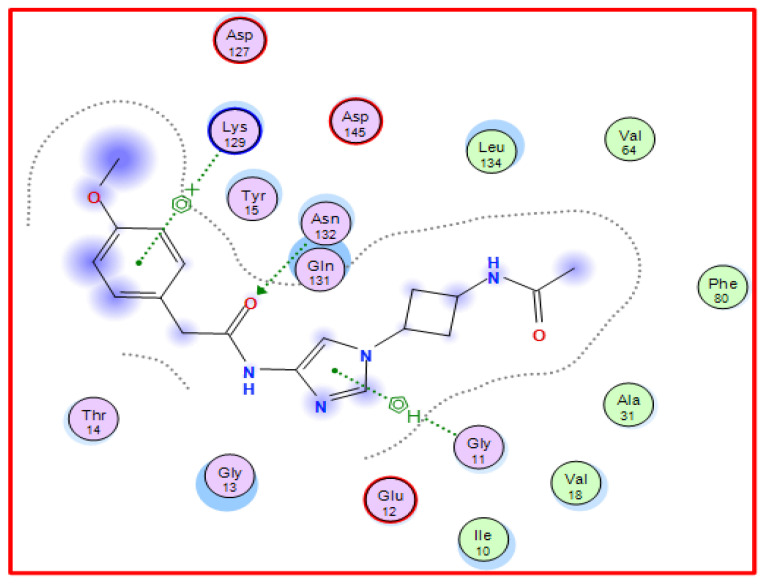
2D Binding of EFP (a reference ligand) with CDK-5.

**Figure 3:**
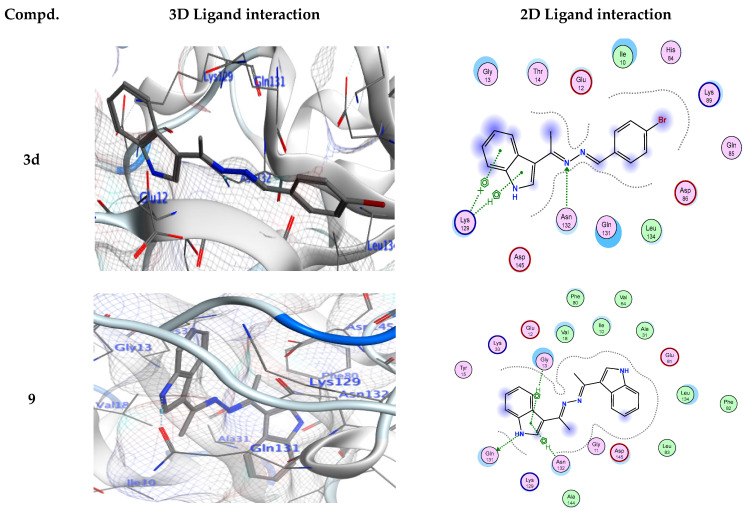

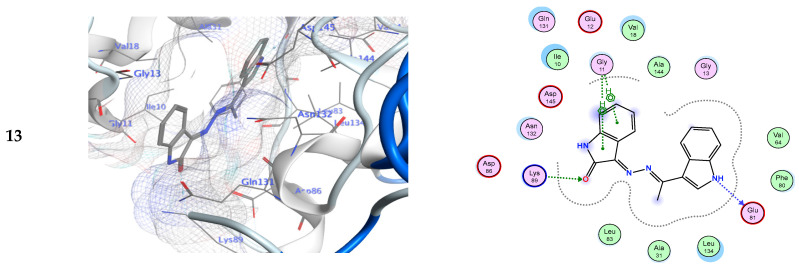
Ligand interactions in 3D and 2D at the 3IG7 receptors’ binding site for derivatives **3d**, **9**, and **13**.

The EFP (*N*-{1-[cis-3-(acetylamino)cyclobutyl]-1*H*-imidazol-4-yl}-2- (4-methoxy phenyl)acetamide) is known to inhibit the enzyme CDK-5 as it exhibits significant interactions with the CDK-5 enzyme’s active site residues [48,49] (Figure 2). Consequently, molecular docking was exploited to stimulate the binding affinities of the synthesized derivatives showing potential anti-tumor activities by locating its structure into the binding site of CDK-5 enzyme (PDB 3ig7) (obtained from the PDB repository at https://www.rcsb.org (accessed on 30 January 2023) with docking data using EFP as a reference ligand) [48,49]. The results of molecular docking simulations indicated that derivatives **3a**–**h**, **7**, **9**, **11**, and **13** exhibited binding energy values between −8.34 to −7.18 kcal/mol. The negative free energy values pertain to the spontaneity of binds (Table 3, Figure 3 and Figure S1).

As shown in Table 3, derivative **3d** was tightly bound into CDK-5 enzyme through one hydrogen bond acceptor via its nitrogen of hydrazinylidene moiety with Asn132. The docking study revealed that derivatives **3e**, **3f**, **3h**, and **13** showed the hydrogen bond donor between NH of these ligands and Glu81 from the enzyme. Additionally, derivative **3f** shows an additional another *H*-bond donor between the oxygen atom of phenol ring and Asp86. Furthermore, derivative **3h** shows another additional *H*-bond acceptor between the chloro atom of the ligand and Lys89. A 3D simulation of the binding mode of derivative **3g** in CDK-5 enzyme showed two *H*-bonds: one *H*-bond donor between the oxygen of the phenol skeleton and Gln131, and another *H*-bond acceptor between the nitrogen of the hydrazinylidene moiety with Lys33, respectively. Due to a strong hydrogen bond donor between the NH of the ligand and Gln131 from the enzyme in addition to another mode of interaction known as ligand exposure, derivative **9** had the highest binding energy to the CDK-5 enzyme (−8.34 Kcal/mol), according to the docking studies.

Alternatively, compound **13** also exhibited a potential inhibitory action for the CDK-5 enzyme (−8.06 Kcal/mol) via a. In addition, there is another hydrogen bond acceptor between the amino acid Lys89 and the carbonyl of the indolin-2-one ring. The role of hydrogen bonds in establishing out the ligand binding specificity [64,65], and its significance for biomacromolecules’ properties and structures has been defined theoretically and experimentally [64]. The results indicated that the comparable positions and orientations within the investigated ligands assumed the binding site of the target enzyme. Thus, the outcomes of molecular docking simulation results indicated that the synthesized derivatives possess higher binding energies than the standard inhibitor (EFP).

#### Drug-Likeness and Oral Bioavailability Analysis of the Most Potent Compounds and Standard

In the early stages of drug discovery, pharmacokinetic property analysis of prospective drugs is essential. According to Lipinski and his team [66], drug-like compounds must obey the rule of five (RO5), i.e., molecular weight (MW) ≤ 500 Da, number of hydrogen bond donor (HBD’s) ≤ 5, and number of hydrogen bond acceptor (HBAs) ≤ 10. The values of the most powerful chemicals are within the permitted range as specified in the RO5, as seen in Tables S1–S3. The polarity of the identified derivatives and reference drug was examined using the total polarity surface area (TPSA), whose acceptable value is between 20 and 130. It is interesting to note that the chosen anticancer and standard have a high likelihood of being absorbed in the human intestine, a major pharmacokinetic characteristic in the development of new drugs. Additionally, the metabolic activities of the chosen drug candidates were predicted using microsomal enzymes (cytochrome P450 inhibitors). All of the chosen drugs and standards do not block any of the cytochrome P450 enzymes, which improve their metabolism as possible therapeutic drugs [67].

## 3. Experimental Section

### 3.1. Chemistry

#### 3.1.1. Materials and Methods

The reported synthetic approach did not include any further purification of any of the reagents or solvents used. Distilled water was used in all experiments. Using Merck silica gel GF254 plates, analytical thin-layer chromatography (TLC) was carried out. The melting points were determined using the Gallenkamp equipment (Shanghai Jiahang Instruments Co., Shanghai, China). The IR spectra (KBr) were obtained using an FTIR spectrometer, Thermo Scientific Nicolet iS10 (SpectraLab Scientific Inc., Markham, ON L3R 3V6, Canada). The ^1^H NMR spectra was detected in DMSO-*d*_6_ using a JEOL’s NMR spectrometer (500 MHz). The mass analysis was performed using a Thermo Scientific GC/MS model ISQ and/or an Agilent LC-MSD IQ Infinity II 1260 ((SpectraLab Scientific Inc., Markham, ON L3R 3V6, Canada). Elemental analysis (C, H, and N) were executed with the Perkin-Elmer 2400 apparatus (Elementar Analysensysteme GmbH, Langenselbold, Germany).

##### General Procedure for the Synthesis of Hydrazones **3a**–**j, 5a,b, 7, 9, 11** and **13**

**Method A:** a mixture of (E)-3-(1-hydrazineylideneethyl)-1*H*-indole **1** (1 mmol, 0.173 g) and the appropriately substituted aldehyde **2a**–**j** or ketone, specifically, acetophenones **4a**,**b**, 2-acetylthiophene **6**, 3-acetylindole **8**, or 3-acetycoumarin **10**, or isatin **12** (1 mmol for each) in acetic acid (20 mL) was refluxed for 3–5 h. (monitored by TLC). The produced precipitate was separated by filtering, followed by washing with methanol, drying, and recrystallization from the appropriate solvent to produce, in turn, products **3a**–**j**, **5a**,**b**, **7**, **9**–**11**, and **13**.

**Method B:** one drop of acetic acid was added to a mixture of (E)-3-(1-hydrazine ylideneethyl)-1*H*-indole **1** (1 mmol, 0.173 g) and the substituted aldehyde **2a**–**j** or ketone, specificall, acetophenones **4a**,**b**, 2-acetylthiophene **6**, 3-acetylindole **8**, or 3-acetycoumarin **10** or isatin **12** (1 mmol for each). Then the mixture was mashed with a pounder in a mortar at 25 °C. When the reaction progresses, the mixture turns into a pasty mixture, which finally solidifies for 20–30 min on completion. TLC (EtOAc: n-hexane, 1:1, *v*/*v*) was used to monitor reaction progress. To get products **3a**–**j**, **5a**,**b**, **7**, **9**–**11**, or **13**, the reaction mixture was drained and filtered, and the impure compounds were purified by recrystallization from the suitable solvent. The spectrum data and physical constants for the compounds are listed below:

*3-((E)-1-(((E)-Benzylidene)hydrazineylidene)ethyl)-1H-indole* (**3a**)

Yellow solid (85%); m.p. 189–191 °C (EtOH); IR (KBr): *v* 3394 (NH), 3038, 2929 (CH), 1593 (C=N) cm^−1^; ^1^H-NMR (DMSO-*d*_6_) *δ* = 2.42 (s, 3H, CH_3_), 7.15–8.26 (m, 10H, Ar-H), 8.68 (CH=N), 11.88 (s, 1H, NH) ppm; ^13^C-NMR (DMSO-*d_6_*): *δ* = 16.1 (CH_3_), 112.1, 116.6, 117.1, 120.1, 120.8, 122.6, 123.6, 125.6, 129.3, 131.4, 137.7, 158.3 (Ar-C and C=N) ppm; MS, *m*/*z* (%) 261 (M^+^, 19). Anal. Calcd. for C_17_H_15_N_3_ (261.33): C, 78.13; H, 5.79; N, 16.08. Found C, 78.01; H, 5.62; N, 16.00%.

*3-((E)-1-(((E)-4-Methoxybenzylidene)hydrazineylidene)ethyl)-1H-indole* (**3b**)

Yellow solid (80%); m.p. 283–285 °C (DMF); IR (KBr): *v* 3394 (NH), 3044, 2925 (CH), 1588 (C=N) cm^−1^; ^1^H-NMR (DMSO-*d*_6_) *δ* = 2.55 (s, 3H, CH_3_), 3.79 (s, 3H, OCH_3_), 7.00–8.59 (m, 9H, Ar-H), 8.67 (CH=N), 11.63 (s, 1H, NH) ppm; MS, *m*/*z* (%) 291 (M^+^, 17). Anal. Calcd. for C_18_H_17_N_3_O (291.35): C, 74.20; H, 5.88; N, 14.42. Found C, 74.06; H, 5.69; N, 14.31%.

*3-((E)-1-(((E)-4-Chlorobenzylidene)hydrazineylidene)ethyl)-1H-indole* (**3c**)

Yellow solid (87%); m.p. 252–254 °C (DMF); IR (KBr): *v* 3394 (NH), 3049, 2938 (CH), 1587 (C=N) cm^−1^; ^1^H-NMR (DMSO-*d*_6_) *δ* = 2.53 (s, 3H, CH_3_), 7.14–8.47 (m, 9H, Ar-H), 8.64 (CH=N), 11.46 (s, 1H, NH) ppm; MS, *m*/*z* (%) 297 (M^+^+2, 9), 295 (M^+^, 30). Anal. Calcd. for C_17_H_14_ClN_3_ (295.77): C, 69.04; H, 4.77; N, 14.21. Found C, 68.91; H, 4.54; N, 14.07%.

*3-((E)-1-(((E)-4-Bromobenzylidene)hydrazineylidene)ethyl)-1H-indole* (**3d**)

Yellow solid (89%); m.p. 259–261 °C (DMF); IR (KBr): *v* 3394 (NH), 3042, 2922 (CH), 1588 (C=N) cm^−1^; ^1^H-NMR (DMSO-*d*_6_) *δ* = 2.53 (s, 3H, CH_3_), 7.14–7.91 (m, 9H, Ar-H), 8.47 (CH=N), 11.47 (s, 1H, NH) ppm; ^13^C-NMR (DMSO-*d_6_*): *δ* = 15.9 (CH_3_), 112.1, 116.0, 120.8, 122.6, 123.2, 124.9, 128.2, 129.2, 129.3, 133.7, 133.8, 137.6, 146.2, 151.7 (Ar-C and C=N) ppm; MS, *m*/*z* (%) 342 (M^+^+2, 19), 340 (M^+^, 21). Anal. Calcd. for C_17_H_14_BrN_3_ (340.22): C, 60.02; H, 4.15; N, 12.35. Found C, 59.88; H, 4.04; N, 12.18%.

*3-((E)-1-(((E)-4-Nitrobenzylidene)hydrazineylidene)ethyl)-1H-indole* (**3e**)

Brown solid (91%); m.p. 195–197 °C (EtOH); IR (KBr): *v* 3393 (NH), 3045, 2927 (CH), 1595 (C=N) cm^−1^; ^1^H-NMR (DMSO-*d*_6_) *δ* = 2.52 (s, 3H, CH_3_), 7.11–8.32 (m, 9H, Ar-H), 8.47 (CH=N), 11.46 (s, 1H, NH) ppm; MS, *m*/*z* (%) 306 (M^+^, 15). Anal. Calcd. for C_17_H_14_N_4_O_2_ (306.33): C, 66.66; H, 4.61; N, 18.29. Found C, 66.50; H, 4.48; N, 18.15%.

*2-((E)-(((E)-1-(1H-Indol-3-yl)ethylidene)hydrazineylidene)methyl)phenol* (**3f**)

Yellow solid (89%); m.p. 296–298 °C (DMF); IR (KBr): *v* 3394, 3247 (NH and OH), 3044, 2921 (CH), 1593 (C=N) cm^−1^; ^1^H-NMR (DMSO-*d*_6_) *δ* = 2.46 (s, 3H, CH_3_), 6.92–7.65 (m, 9H, Ar-H), 8.17 (CH=N), 8.96 (s, 1H, OH), 11.11 (s, 1H, NH) ppm; MS, *m*/*z* (%) 277 (M^+^, 17). Anal. Calcd. for C_17_H_15_N_3_O (277.33): C, 73.63; H, 5.45; N, 15.15. Found C, 73.52; H, 5.57; N, 15.03%.

*4-((E)-(((E)-1-(1H-indol-3-yl)ethylidene)hydrazineylidene)methyl)phenol* (**3g**)

White solid (92%); m.p. 299–301 °C (DMF); IR (KBr): *v* 3394, 3296 (NH and OH), 3043, 2927 (CH), 1588 (C=N) cm^−1^; ^1^H-NMR (DMSO-*d*_6_) *δ* = 2.54 (s, 3H, CH_3_), 6.83–8.46 (m, 9H, Ar-H), 8.53 (CH=N), 10.04 (s, 1H, OH), 11.48 (s, 1H, NH) ppm; MS, *m*/*z* (%) 277 (M^+^, 100). Anal. Calcd. for C_17_H_15_N_3_O (277.33): C, 73.63; H, 5.45; N, 15.15. Found C, 73.51; H, 5.37; N, 15.00%.

*3-((E)-1-(((E)-2,4-Dichlorobenzylidene)hydrazineylidene)ethyl)-1H-indole* (**3h**)

Yellow solid (86%); m.p. 253–255 °C (DMF); IR (KBr): *v* 3394 (NH), 3039, 2925 (CH), 1603 (C=N) cm^−1^; ^1^H-NMR (DMSO-*d*_6_) *δ* = 2.46 (s, 3H, CH_3_), 6.70–8.47 (m, 8H, Ar-H), 8.67 (CH=N), 11.69 (s, 1H, NH) ppm; ^13^C-NMR (DMSO-*d_6_*): *δ* = 15.9 (CH_3_), 113.3, 116.5, 117.9, 119.2, 124.9, 125.3, 125.8, 127.0, 129.7, 129.8, 133.1, 141.7, 142.0, 143.5, 154.0, 159.9 (Ar-C and C=N) ppm; MS, *m*/*z* (%) 330 (M^+^, 18). Anal. Calcd. for C_17_H_13_Cl_2_N_3_ (330.21): C, 61.84; H, 3.97; N, 12.73. Found C, 61.70; H, 3.99; N, 12.59%.

*2-((E)-(((E)-1-(1H-Indol-3-yl)ethylidene)hydrazineylidene)methyl)-4-bromophenol* (**3i**)

Yellow solid (81%); m.p. 275–277 °C (DMF); IR (KBr): *v* 3394, 3158 (NH and OH), 3046, 2929 (CH), 1590 (C=N) cm^−1^; ^1^H-NMR (DMSO-*d*_6_) *δ* = 2.46 (s, 3H, CH_3_), 6.91–8.48 (m, 8H, Ar-H), 8.90 (CH=N), 11.10 (s, 1H, OH), 11.47 (s, 1H, NH) ppm; MS, *m*/*z* (%) 358 (M^+^+2, 24), 356 (M^+^, 27). Anal. Calcd. for C_17_H_14_BrN_3_O (356.22): C, 57.32; H, 3.96; N, 11.80. Found C, 57.13; H, 3.88; N, 11.69%.

*2-(4-((E)-(((E)-1-(1H-indol-3-yl)ethylidene)hydrazineylidene)methyl)-3-methoxyphenoxy)-N-(4-bromophenyl)acetamide* (**3j**)

Yellow solid (83%); m.p. 265–267 °C (DMF); IR (KBr): *v* 3392, 3269 (2NH), 3281, 2939 (CH), 1697 (C=O), 1589 (C=N) cm^−1^; ^1^H-NMR (DMSO-*d*_6_) *δ* = 2.52 (s, 3H, CH_3_), 3.84 (s, 3H, OCH_3_), 4.75 (s, 2H, CH_2_), 7.00–8.46 (m, 12H, Ar-H), 8.61 (CH=N), 10.26 (s, 1H, NH), 11.48 (s, 1H, NH) ppm; ^13^C-NMR (DMSO-*d_6_*): *δ* = 15.7 (CH_3_), 56.2 (OCH_3_), 66.2 (CH_2_), 103.0, 109.8, 114.0, 114.1, 115.8, 121.9, 122.3, 125.9, 126.1, 128.7, 129.9, 130.2, 132.1, 138.3, 142.8, 149.6, 149.8, 150.0, 158.2 (Ar-C and C=N), 167.1 (C=O) ppm; MS, *m*/*z* (%) 521 (M^+^+2, 15), 519 (M^+^, 16). Anal. Calcd. for C_26_H_23_BrN_4_O_3_ (519.40): C, 60.12; H, 4.46; N, 10.79. Found C, 60.04; H, 4.31; N, 10.65%.

*3-((E)-1-(((E)-1-Phenylethylidene)hydrazineylidene)ethyl)-1H-indole* (**5a**)

Yellow solid (87%); m.p. 189–191 °C (DMF); IR (KBr): *v* 3394 (NH), 3030, 2926 (CH), 1588 (C=N) cm^−1^; ^1^H-NMR (DMSO-*d*_6_) *δ* = 2.23 (s, 3H, CH_3_), 2.54 (s, 3H, CH_3_), 7.13–8.48 (m, 10H, Ar-H), 11.49 (s, 1H, NH) ppm; ^13^C-NMR (DMSO-*d_6_*): *δ* = 14.1 (CH_3_), 15.7 (CH_3_), 112.2, 116.2, 117.1, 117.1, 118.7, 120.1, 121.5, 124.3, 131.4, 131.7, 133.7, 140.2, 142.3, 149.4 (Ar-C and C=N) ppm; MS, *m*/*z* (%) 275 (M^+^, 49). Anal. Calcd. for C_18_H_17_N_3_ (275.36): C, 78.52; H, 6.22; N, 15.26. Found C, 78.64; H, 6.14; N, 15.12%.

*3-((E)-1-(((E)-1-(4-Bromophenyl)ethylidene)hydrazineylidene)ethyl)-1H-indole* (**5b**)

Yellow solid (80%); m.p. 183–185 °C (DMF); IR (KBr): *v* 3413 (NH), 3053, 2920 (CH), 1590 (C=N) cm^−1^; ^1^H-NMR (DMSO-*d*_6_) *δ* = 2.22 (s, 3H, CH_3_), 2.53 (s, 3H, CH_3_), 7.11–7.92 (m, 9H, Ar-H), 11.49 (s, 1H, NH) ppm; MS, *m*/*z* (%) 356 (M^+^+2, 14), 354 (M^+^, 16). Anal. Calcd. for C_18_H_16_BrN_3_ (354.25): C, 61.03; H, 4.55; N, 11.86. Found C, 61.00; H, 4.43; N, 11.70%.

*3-((E)-1-(((E)-1-(2-Thienyl)ethylidene)hydrazineylidene)ethyl)-1H-indole* (**7**)

Yellow solid (88%); m.p. 284–286 °C (EtOH); IR (KBr): *v* 3214 (NH), 2920 (CH), 1600 (C=N) cm^−1^; ^1^H-NMR (DMSO-*d*_6_) *δ* = 2.42 (s, 3H, CH_3_), 2.54 (s, 3H, CH_3_), 7.12–8.48 (m, 8H, Ar-H), 11.39 (s, 1H, NH) ppm; ^13^C-NMR (DMSO-*d_6_*): *δ* = 15.4 (CH_3_), 16.1 (CH_3_), 112.1, 116.6, 120.8, 122.6, 123.6, 125.6, 126.4, 127.8, 128.4, 129.3, 130.4, 137.7, 158.3, 160.5 (Ar-C and C=N) ppm; MS, *m*/*z* (%) 281 (M^+^, 24). Anal. Calcd. for C_16_H_15_N_3_S (281.38): C, 68.30; H, 5.37; N, 14.90. Found C, 68.14; H, 5.25; N, 14.72% [68].

*(1E,2E)-1,2-Bis(1-(1H-indol-3-yl)ethylidene)hydrazine* (**9**)

Yellow solid (82%); m.p. 215–217 °C (EtOH); IR (KBr): *v* 3393 (NH), 3027, 2933 (CH), 1613 (C=N) cm^−1^; ^1^H-NMR (DMSO-*d*_6_) *δ* = 2.55 (s, 6H, 2CH_3_), 7.12–7.17 (m, 4H, Ar-H), 7.42–7.43 (d, 2H, Ar-H), 7.93 (s, 2H, Ar-H), 8.48–8.50 (d, 2H, Ar-H), 11.49 (s, 2H, 2NH) ppm; ^13^C-NMR (DMSO-*d_6_*): *δ* = 16.1 (CH_3_), 112.2, 116.6, 120.9, 122.7, 123.7, 125.70, 129.3, 137.8, 158.4 (Ar-C and C=N) ppm; MS, *m*/*z* (%) 314 (M^+^, 22). Anal. Calcd. for C_20_H_18_N_4_ (314.39): C, 76.41; H, 5.77; N, 17.82. Found C, 76.32; H, 5.53; N, 17.61%.

*3-((E)-1-(((E)-1-(1H-Indol-3-yl)ethylidene)hydrazineylidene)ethyl)-2H-chromen-2-one* (**11**)

Yellow solid (88%); m.p. 181–183 °C (EtOH); IR (KBr): *v* 3393 (NH), 3049, 2922 (CH), 1723 (C=O), 1588 (C=N) cm^−1^; ^1^H-NMR (DMSO-*d*_6_) *δ* = 2.46 (s, 3H, CH_3_), 2.53 (s, 3H, CH_3_), 7.10–8.48 (m, 10H, Ar-H), 11.48 (s, 1H, NH); ^13^C-NMR (DMSO-*d_6_*): *δ* = 13.1 (CH_3_), 16.0 (CH_3_), 112.2, 115.6, 120.5, 120.6, 120.9, 121.9, 122.7, 122.9, 123.2, 123.6, 125.3, 129.6, 134.8, 135.0, 137.7, 143.6, 144.5, 159.3 (Ar-C and C=N), 165.0 (C=O) ppm; MS, *m*/*z* (%) 343 (M^+^, 13). Anal. Calcd. for C_21_H_17_N_3_O_2_ (343.39): C, 73.45; H, 4.99; N, 12.24. Found C, 73.33; H, 4.91; N, 12.05%.

*(E)-3-(((E)-1-(1H-Indol-3-yl)ethylidene)hydrazineylidene)indolin-2-one* (**13**)

Orange solid (84%); m.p. 261–263 °C (DMF); IR (KBr): *v* 3394 (NH), 3028, 2930 (CH), 1692 (C=O), 1589 (C=N) cm^−1^; ^1^H-NMR (DMSO-*d*_6_) *δ* = 2.46 (s, 3H, CH_3_), 6.81–8.26 (m, 9H, Ar-H), 9.54 (s, 1H, NH), 10.64 (s, 1H, NH) ppm; ^13^C-NMR (DMSO-*d_6_*): *δ* = 15.9 (CH_3_), 112.1, 114.2, 115.9, 120.8, 122.6, 123.2, 123.9, 124.9, 128.2, 130.0, 134.0, 137.6, 141.9, 143.0, 146.2, 155.8 (Ar-C and C=N), 170.7 (C=O) ppm; MS, *m*/*z* (%) 302 (M^+^, 37). Anal. Calcd. for C_18_H_14_N_4_O (302.34): C, 71.51; H, 4.67; N, 18.53. Found C, 71.35; H, 4.50; N, 18.39%.

### 3.2. Cytotoxic Activity

The cytotoxicity of freshly synthesized azines was investigated against HCT-116, MCF-7, and HepG2 cells using the MTT test over a 24-h incubation period [69,70].

Mammalian cell line: HCT-116, MCF-7, and HepG2 cells were obtained from the VACSERA Tissue Culture Unit in Cairo, Egypt.

### 3.3. Docking Study

All information about the docking study is explained in the Supplementary Materials file [59,71,72,73,74].

## 4. Conclusions

The present study disclosed the green synthesis of 16 new benzaldazine and ketazine derivatives through the reaction of 3-(1-hydrazineylideneethyl)-*1H*-indole with various carbonyl compounds (aldehydes and ketones). Elemental and spectral analyses were used to confirm the structures of the produced derivatives. By using in vitro and in silico docking studies, all the synthesized derivatives were found to be efficient in inhibiting human colon cancer, human breast adenocarcinoma, and hepatocellular carcinoma cell lines. Molecular docking has proceeded for the 12 compounds **3a**–**h**, **7**, **9**, **11**, and **13** on CDK-5 enzyme in an attempt to show their manner of action as anticancer drugs. The complete analysis of the structure–activity connection, advancements, and pharmacological activity of substituted hydrazineylidene-*1H*-indole derivatives stated and elucidated in the article will aid researchers generating innovative, powerful, and safe anticancer candidates in the future.

## Figures and Tables

**Table 1:** Comparison of the traditional heating and grinding method for synthesizing aldazines **3a**–**j**.

Product No.	Conventional		Grinding Technique	
	Time (h)	Yield (%)	Time (min)	Yield (%)
**3a**	4	70	17	90
**3b**	3	71	20	91
**3c**	3	69	19	88
**3d**	4	75	19	87
**3e**	5	72	18	88
**3f**	4	71	17	89
**3g**	5	73	20	91
**3h**	4	74	22	92
**3i**	3	70	21	89
**3j**	3	72	20	91

**Table 2:** Cytotoxic activity of synthesized derivatives against human tumor cell lines.

Derivatives	In Vitro Cytotoxicity IC_50_ ± S. D (µM)		
	HCT-116	HePG-2	MCF-7
**3a**	44.24 ± 2.30	35.38 ± 2.30	53.64 ± 1.84
**3b**	52.28 ± 3.47	45.29 ± 3.57	69.28 ± 4.16
**3c**	8.15 ± 2.00	8.41 ± 2.26	13.36 ± 1.37
**3d**	4.27 ± 2.41	4.83 ± 1.94	7.93 ± 1.30
**3e**	19.07 ± 2.15	19.78 ± 2.72	17.02 ± 2.12
**3f**	33.81 ± 3.83	28.27 ± 2.30	68.14 ± 3.16
**3g**	29.29 ± 2.93	23.17 ± 2.28	57.45 ± 3.38
**3h**	5.11 ± 1.75	5.03 ± 0.93	8.39 ± 2.19
**7**	29.38 ± 2.93	31.71 ± 3.19	50.34 ± 2.61
**9**	4.42 ± 0.96	4.09 ± 0.85	6.19 ± 2.37
**11**	10.18 ± 2.37	9.05 ± 2.39	13.27 ± 3.04
**13**	6.01 ± 1.39	4.87 ± 1.32	7.91 ± 2.37
Doxorubicin	5.23 ± 0.29	4.50 ± 0.20	4.17 ± 0.20

**Table 3:** Docking score (kcal/mol), no. of *H*-bonding, and no. Of arene interaction formed between synthesized derivatives **3a**–**h**, **7**, **9**, **11**, and **13** with 3IG7 receptors when compared to native co-crystallized ligand EFP.

Derivative No.	Docking Score (kcal/mol)	No. of Hydrogen Bonding	Affinity Bond Length (Å from Hydrogen Bond)	No. of Arene Interaction
**3a**	−7.49	-	-	1 (π-H) [Glu12]
**3b**	−7.18	-	-	-
**3c**	−7.61	-	-	1 (π-H) [Glu12]
**3d**	−7.82	1 (Asn132)	2.93	1 (π-H) [Lys129]
				1 (π-cation) [Lys129]
**3e**	−7.44	1 (Glu81)	2.93	1 (π-H) [Gly11]
**3f**	−7.31	1 (Glu81)	2.92	1 (π-H) [Gly11]
		1 (Asp86)	3.61	
**3g**	−7.36	1 (Gln131)	2.91	-
		1 (Lys33)	2.92	
**3h**	−7.75	1 (Glu81)	2.85	-
		1 (Lys89)	2.91	
**7**	−7.27	-	-	1 (π-H) [Val18]
				1 (π-H) [Lys129]
**9**	−8.34	1 (Gln131)	3.04	1 (π-H) [Gly13]
				1 (π-H) [Asn132]
**11**	−7.53	-	-	1 (π-H) [Gln85]
**13**	−8.06	1 (Glu81)	3.03 3.04	2 (π -H) [Gly11]
		1 (Lys89)		
Doxorubicin	−7.04	1 (Asn132)	3.04	1 (π-H) [Gly11]
				1 (π-cation) [Lys129]

## Data Availability

The data presented in this study are available on request.

## Supplementary Materials

The following supporting information can be downloaded at: https://www.mdpi.com/1420-3049/28/9/3869/s1, Figure S1: ligand interactions in 3D and 2D at the 3IG7 receptors’ binding site for derivatives **3a**–**h**, **7**, **9**, **11** and **13**; Table S1: Computed values for prediction parameters of derivative **9**; Table S2: Computed values for prediction parameters of derivative **3d**; Table S3: Computed values for prediction parameters of Doxorubicin; Section 3.2. Docking study; and some ^1^H-, ^13^C-NMR and Mass spectra of the synthesized compounds

## Sample Availability

Samples of the compounds **3a**–**j**, **5a**,**b**, **7**, **9**, **11** and **13** are available from the authors.

## References

[1] Siegel R.L., Miller K.D., Fedewa S.A., Ahnen D.J., Meester R.G., Barzi A., Jemal A. (2017). Colorectal cancer statistics. CA Cancer J. Clin..

[2] Bray F., Ferlay J., Soerjomataram I., Siegel R.L., Torre L.A., Jemal A. (2018). Global cancer statistics 2018: GLOBOCAN estimates of incidence and mortality worldwide for 36 cancers in 185 countries. CA Cancer J. Clin..

[3] Akhtar J., Khan A.A., Ali Z., Haider R., Shahar Yar M. (2017). Structure-activity relationship (SAR) study and design strategies of nitrogen-containing heterocyclic moieties for their anticancer activities. Eur. J. Med. Chem..

[4] Fadeyi O.O., Adamson S.T., Myles E.L., Okoro C.O. (2008). Novel fluorinated acridone derivatives. Part 1: Synthesis and evaluation as potential anticancer agents. Bioorg. Med. Chem. Lett..

[5] Sivaprasad G., Perumal P.T., Prabavathy V.R., Mathivanan N. (2006). Synthesis and anti-microbial activity of pyrazolylbisindoles-promising anti-fungal compounds. Bioorg. Med. Chem. Lett..

[6] Leboho T.C., Michael J.P., van Otterlo W.A., van Vuuren S.F., de Koning C.B. (2009). The synthesis of 2- and 3-aryl indoles and 1, 3, 4, 5-tetrahydropyrano [4, 3-b]indoles and their antibacterial and antifungal activity. Bioorg. Med. Chem. Lett..

[7] Hu W., Guo Z., Yi X., Guo C., Chu F., Cheng G. (2003). Discovery of 2-phenyl-3-sulfonylphenyl- indole derivatives as a new class of selective COX-2 inhibitors. Bioorg. Med. Chem..

[8] Battaglia S., Boldrini E., Da Settimo F., Dondio G., La Motta C., Marini A.M., Primofiore G. (1999). Indole amide derivatives: Synthesis, structure–activity relationships and molecular modelling studies of a new series of histamine H 1-receptor antagonists. Eur. J. Med. Chem..

[9] Karaaslan C., Kadri H., Coban T., Suzen S., Westwell A.D. (2013). Synthesis and antioxidant properties of substituted 2-phenyl-1*H*-indoles. Bioorg. Med. Chem. Lett..

[10] Li Y.-Y., Wu H.-S., Tang L., Feng C.-R., Yu J.-H., Li Y., Yang Y.-S., Yang B., He Q.-J. (2007). The potential insulin sensitizing and glucose lowering effects of a novel indole derivative in vitro and in vivo. Pharmacol. Res..

[11] Abdel-Gawad H., Mohamed H.A., Dawood K.M., Badria F.A.-R. (2010). Synthesis and antiviral activity of new indole-based heterocycles. Chem. Pharm. Bull..

[12] Ghanei-Nasab S., Khoobi M., Hadizadeh F., Marjani A., Moradi A., Nadri H., Emami S., Foroumadi A., Shafiee A. (2016). Synthesis and anticholinesterase activity of coumarin-3-carboxamides bearing tryptamine moiety. Eur. J. Med. Chem..

[13] Greenwell M., Rahman P.K.S.M. (2015). Medicinal Plants: Their Use in Anticancer Treatment. Int. J. Pharm. Sci. Res..

[14] Abdelhamid A.O., Gomha S.M., Abdelriheem N.A., Kandeel S.M. (2016). Synthesis of new 3-heteroarylindoles as potential anticancer agents. Molecule.

[15] MacDonough M.T., Strecker T.E., Hamel E., Hall J.J., Chaplin D.J., Trawick M.L., Pinney K.G. (2013). Synthesis and biological evaluation of indole-based, anti-cancer agents inspired by the vascular disrupting agent 2-(3′-hydroxy-4′-methoxyphenyl)-3-(3″,4″,5″-trimethoxybenzoyl)-6-methoxyindole (OXi8006). Bioorg. Med. Chem..

[16] Akkoç M.K., Yüksel M.Y., Durmaz Đ., Atalay R.Ç. (2012). Design, synthesis, and biological evaluation of indole-based 1, 4-disubstituted piperazines as cytotoxic agents. Turk. J. Chem..

[17] Kumar D., Kumar N.M., Noel B., Shah K. (2012). A series of 2-arylamino-5-(indolyl)-1, 3, 4-thiadiazoles as potent cytotoxic agents. Eur. J. Med. Chem..

[18] Queiroz M.-J.R., Abreu A.S., Carvalho M.S.D., Ferreira P.M., Nazareth N., Nascimento M.S.-J. (2008). Synthesis of new heteroaryl and heteroannulated indoles from dehydrophenylalanines: Antitumor evaluation. Bioorg. Med. Chem..

[19] Zhang F., Zhao Y., Sun L., Ding L., Gu Y., Gong P. (2011). Synthesis and anti-tumor activity of 2-amino-3- cyano-6-(1*H*-indol-3-yl)-4-phenylpyridine derivatives in vitro. Eur. J. Med. Chem..

[20] Ahmad A., Sakr W.A., Rahman K.M. (2010). Anticancer properties of indole compounds: Mechanism of apoptosis induction and role in chemotherapy. Curr. Drug Targets..

[21] Veena K., Ramaiah M., Shashikaladevi K., Avinash T.S., Vaidya V.P. (2011). Synthesis and antimicrobial activity of asymmetrical azines derived from naphtho[2,1-b]furan. J. Chem. Pharm. Res..

[22] Liang C., Xia J., Lei D., Li X., Yao Q., Gao J. (2014). Synthesis, in vitro and in vivo antitumor activity of symmetrical bis-Schiff base derivatives of isatin. Eur. J. Med. Chem..

[23] Danish I.A., Prasad K.R. (2004). Synthesis and characterisation of N, N’-biscarbazolyl azine and N, N′-carbazolyl Hydrazine derivatives and their antimicrobial studies. Acta Pharm..

[24] Gul H.I., Calis U., Vepsalainen J. (2004). Synthesis of some mono-Mannich bases and corresponding azine derivatives and evaluation of their anticonvulsant activity. Arzneim.-Forsch.-Drug Res..

[25] Eberle M., Farooq S., Jeanguenat A., Mousset D., Steiger A., Trah S., Zambach W., Rindlisbacher A. (2003). Azine derivatives as a new generation of insect growth regulators. Chimia.

[26] Dimmock J.R., Kumar P., Quail J.W., Pugazhenthi U., Yang J., Chen M., Reid R.S., Allen T.M., Kao G.Y., Cole S.P.C. (1995). Synthesis and Cytotoxic Evaluation of Some Styryl Ketones and Related Compounds. Eur. J. Med. Chem..

[27] Gul H.I., Gul M., Vepsälainen J., Erciyas E., Hänninen O. (2003). Cytotoxicity of Some Azines of Acetophenone Derived Mono-Mannich Bases against Jurkat Cells. Biol. Pharm. Bull..

[28] Haider N., Kabicher T., Käferböck J., Plenk A. (2007). Synthesis and *in-vitro* antitumor activity of 1-[3- (indol-1-yl) prop-1-yn-1-yl] phthalazines and related compounds. Molecules.

[29] Sachdev H., Mathur J., Guleria A. (2020). Indole derivatives as potential anticancer agents: A review. J. Chil. Chem. Soc..

[30] El-Din N.S., Barseem A. (2016). Synthesis, bioactivity and docking study of some new indole-hydrazone derivatives. J. Appl. Pharm..

[31] Kumari A., Singh R.K. (2019). Medicinal chemistry of indole derivatives: Current to future therapeutic prospectives. Bioorg. Chem..

[32] Mousavi H.A. (2021). comprehensive survey upon diverse and prolific applications of chitosan-based catalytic systems in one-pot multi-component synthesis of heterocyclic rings. Int. J. Bio. Macromol..

[33] Jimenez-Gonzalez C., Lund C. (2022). Green metrics in pharmaceutical development. Curr. Opin. Green Sustain. Chem..

[34] Koenig S.G., Bee C., Borovika A., Briddell C., Colberg J., Humphery G.R., Kopach M.E., Martinez I., Nambiar S., Plummer S.V. (2019). Green chemistry continuum for a robust and sustainable active pharmaceutical ingredient supply chain. ACS Sustain. Chem. Eng..

[35] Karhale S., Survase D., Bhat R., Ubale P., Helavi V. (2017). A practical and green protocol for the synthesis of 2,3-dihydroquinazolin-4(1*H*)-ones using oxalic acid as organocatalyst. Res. Chem. Intermed..

[36] Gomha S.M., Badrey M.G., Arafa W.A.A. (2016). DABCO-catalyzed green synthesis of thiazole and 1,3-thiazine derivatives linked to benzofuran. Heterocycles.

[37] Khalil K.D., Riyadh S.M., Gomha S.M., Ali I. (2019). Synthesis, characterization and application of copper oxide chitosan nanocomposite for green regioselective synthesis of [1, 2, 3]triazoles. Int. J. Biol. Macromol..

[38] Alshabanah L.A., Al-Mutabagani L.A., Gomha S.M., Ahmed H.A. (2022). Three-component synthesis of some new coumarin derivatives as anti-cancer agents. Front. Chem..

[39] Gomha S.M., Abdelaziz M.R., Abdel-aziz H.M., Hassan S.A. (2017). Green synthesis and molecular docking of thiazolyl-thiazole derivatives as potential cytotoxic agents. Mini-Rev. Med. Chem..

[40] Gomha S.M., Muhammad Z.A., Abdel-aziz H.M., Matar I.K., El-Sayed A.A. (2020). Green synthesis, molecular docking and anticancer activity of novel 1,4-dihydropyridine-3,5- dicarbohydrazones under grind-stone chemistry. Green Chem. Lett. Rev..

[41] Zhou B., Liu Z., Qu W., Yang R., Lin X., Yan S., Lin J. (2014). An environmentally benign, mild, and catalyst-free reaction of quinones with heterocyclic ketene aminals in ethanol: Site-selective synthesis of rarely fused [1,2-a]indolone derivatives via an unexpected anti-Nenitzescu strategy. Green Chem..

[42] Gomha S.M., Riyadh S.M. (2009). Synthesis of triazolo[4,3-b][1,2,4,5]tetrazines and triazolo[3,4-b][1,3,4]thiadiazines using chitosan as ecofriendly catalyst under microwave irradiation. Arkivoc.

[43] Kiyani H., Ghorbani F. (2016). Expeditious green synthesis of 3,4-disubstituted isoxazole-5(4*H*)-ones catalyzed by nano-MgO. Res. Chem. Intermed..

[44] Pagadala N.S., Syed K., Tuszynski J. (2017). Software for molecular docking: A review. Biophys Rev..

[45] Silva D.R., Sardi J.D.C.O., Freires I.A., Silva A.C.B., Rosalen P.L. (2019). In silico approaches for screening molecular targets in Candida albicans: A proteomic insight into drug discovery and development. Eur. J. Pharmacol..

[46] Karthik L., Kumar G., Keswani T., Bhattacharyya A., Chandar S.S., Bhaskara Rao K.V. (2014). Protease inhibitors from marine actinobacteria as a potential source for antimalarial compound. PLoS ONE.

[47] McInnes C. (2008). Progress in the evaluation of CDK inhibitors as anti-tumor agents. Drug Discov. Today.

[48] Helal C.J., Kang Z., Lucas J.C., Gant T., Ahlijanian M.K., Schachter J.B., Richter K.E.G., Cook J.M., Menniti F.S., Kelly K. (2009). Potent and cellularly active 4-aminoimidazole inhibitors of cyclin-dependent kinase 5/p25 for the treatment of Alzheimer’s disease. Bioorg. Med. Chem. Lett..

[49] Gouda M.A.S., Salem M.A.I., Mahmoud N.F.H. (2020). 3D-pharmacophore study molecular docking and synthesis of pyrido [2, 3-d] pyrimidine-4(1H)dione derivatives with in vitro potential anticancer and antioxidant activities. J. Heterocycl. Chem..

[50] Gomha S.M., Abdalla M.A., Abdelaziz M., Serag N. (2016). Eco-friendly *one-pot* synthesis and antiviral evaluation of pyrazolyl pyrazolines of medicinal interest. Turk. J. Chem..

[51] Gomha S.M., Muhammad Z.A., Abdel-aziz M.R., Abdel-aziz H.M., Gaber H.M., Elaasser M.M. (2018). One pot synthesis of new thiadiazolyl-pyridines as anticancer and antioxidant agents. J. Heterocycl. Chem..

[52] Abu-Melha S., Edrees M.M., Riyadh S.M., Abdelaziz M.R., Elfiky A.A., Gomha S.M. (2020). Clean grinding technique: A facile synthesis and in silico antiviral activity of hydrazones, pyrazoles, and pyrazines bearing thiazole moiety against SARS-CoV-2 main protease (Mpro). Molecules.

[53] Abdallah M.A., Riyadh S.M., Abbas I.M., Gomha S.M. (2005). Synthesis and biological activities of 7-arylazo-7*H*-pyrazolo [5,1-*c*][1,2,4]triazolo-6(5*H*)-ones and 7-arylhydrazono- 7*H*-[1,2,4]triazolo [3,4-*b*][1,3,4]thiadiazines. J. Chin. Chem. Soc..

[54] Abbas I.M., Riyadh S.M., Abdallah M.A., Gomha S.M. (2006). A novel route to tetracyclic fused tetrazines and thiadiazines. J. Heterocycl. Chem..

[55] Gomha S.M. (2009). A facile one-pot synthesis of 6,7,8,9-tetrahydrobenzo[4,5]thieno[2,3-*d*]-1,2,4-triazolo[4,5-*a*]pyrimidin-5-ones. Monatsh. Chem..

[56] Yaccoubi F., El-Naggar M., Abdelrazek F.M., Gomha S.M., Farghaly M.S., Abolibda T.Z., Ali L.A., Abdelmonsef A.H. (2022). Pyrido-pyrimido-thiadiazinones: Green synthesis, molecular docking studies and biological investigation as obesity inhibitors. J. Taibah Univ. Sci..

[57] Abolibda T.Z., Fathalla M., Farag B., Zaki M.E.A., Gomha S.M. (2023). Synthesis and molecular docking of some novel 3-thiazolyl-coumarins as inhibitors of VEGFR-2 kinase. Molecules.

[58] Gomha S.M., Riyadh S.M., Alharbi R.A.K., Zaki M.E.A., Abolibda T.Z., Farag B. (2023). Green route synthesis and molecular docking of azines using cellulose sulfuric acid under microwave irradiation. Crystals.

[59] Alghamdi A., Abouzied A.S., Alamri A., Anwar S., Ansari M., Khadra I., Zaki Y.H., Gomha S.M. (2023). Synthesis, molecular docking and dynamic simulation tar-geting main protease (mpro) of new thiazole clubbed pyridine scaffolds as potential COVID-19 inhibitors. Curr. Issues Mol. Biol..

[60] Kassem E.M., Mandour A.H. (1999). Some 3-indole derivatives with evaluation of their antimicrobial activity. Egypt. J. Chem..

[61] Yin L.J., bin Ahmad Kamar A.K.D., Fung G.T., Liang C.T., Avupati V.R. (2022). Review of anticancer potentials and structure-activity relationships (SAR) of rhodanine derivatives. Biomed. Pharmacother..

[62] Dong H., Liu J., Liu X., Yu Y., Cao S. (2017). Molecular docking and QSAR analyses of aromatic heterocycle thiosemicarbazone analogues for finding novel tyrosinase inhibitors. Bioorg. Chem..

[63] Dong H., Liu J., Liu X., Yu Y., Cao S. (2018). Combining molecular docking and QSAR studies for modeling the anti-tyrosinase activity of aromatic heterocycle thiosemicarbazone analogues. J. Mol. Struct..

[64] Meyer M., Wilson P., Schomburg D. (1996). Hydrogen bonding and molecular surface shape complementarity as a basis for protein docking. J. Mol. Biol..

[65] Nallal V.U.M., Padmini R., Ravindran B., Chang S.W., Radhakrishnan R., Almoallim H.S.M., Alharbi S.A., Razia M. (2021). Combined in vitro and in silico approach to evaluate the inhibitory potential of an underutilized allium vegetable and its pharmacologically active compounds on multidrug resistant Candida species. Saudi J. Biol. Sci..

[66] Lipinski C.A. (2004). Lead- and drug-like compounds: The rule-of-five revolution. Drug Discov Today Technol..

[67] Abdul-Hammed M., Adedotun I.O., Falade V.A., Adepoju A.J., Olasupo S.B., Akinboade M.W. (2021). Target-based drug discovery, ADMET profiling and bioactivity studies of antibiotics as potential inhibitors of SARS-CoV-2 main protease (Mpro). Virusdisease.

[68] Sayed A.R., Gomha S.M., Abdelrazek F.M., Farghaly M.S., Hassan S.A., Metz P. (2019). Design, efficient synthesis and molecular docking of some novel thiazolyl-pyrazole derivatives as anticancer agents. BMC Chem..

[69] Gomha S.M., Riyadh S.M., Mahmmoud E.A., Elaasser M.M. (2015). Synthesis and anticancer activity of arylazothiazoles and 1,3,4-thiadiazoles using chitosan-grafted-poly(4-vinylpyridine) as a novel copolymer basic catalyst. Chem. Heterocycl. Compd..

[70] Trivedi M., Vaidya D., Patel C., Prajapati S., Bhatt J. (2020). In silico and in vitro studies to elucidate the role of 1HYN and 1QKI activity in BPA induced toxicity and its amelioration by Gallic acid. Chemosphere.

[71] Gao M., Nie K., Qin M., Xu H., Wang F., Liu L. (2021). Molecular mechanism study on stereo-selectivity of α or β hydroxysteroid dehydrogenases. Crystals.

[72] Hajdúch M., Havlíèek L., Veselý J., Novotný R., Mihál V., Strnad M. (1999). Synthetic cyclin dependent kinase inhibitors: New generation of potent anti-cancer drugs. Drug Resist. Leuk. Lymphoma III.

[73] Labute P. (2009). Function, and Bioinformatics, Protonate3D: Assignment of ionization states and hydrogen coordinates to macromolecular structures. Proteins Struct. Funct. Bioinform..

[74] Kattan S.W., Nafie M.S., Elmgeed G.A., Alelwani W., Badar M., Tantawy M.A. (2020). Molecular docking, anti-proliferative activity and induction of apoptosis in human liver cancer cells treated with androstane derivatives: Implication of PI3K/AKT/mTOR pathway. J. Steroid Biochem. Mol. Biol..

