# Supplementary material for: Mechanochemical Synthesis and Molecular Docking Studies of New Azines Bearing Indole as Anticancer Agents
Source: Molecules. 2023 May 4;28(9):3869. doi: 10.3390/molecules28093869 (PMC10180502; doi:10.3390/molecules28093869)
Supplement: Supplementary file 1 [file molecules-28-03869-s001.zip › molecules-2324218-supplementary.pdf]

Supplementary File

Mechanochemical Synthesis and Molecular Docking Studies of New Azines Bearing Indole as Anticancer Agents

Mohamed S. Ibrahim[1], Basant Farag[2], Jehan Y. Al-Humaidi[3], Magdi E. A. Zaki[4], Maher Fathalla[1,2], Sobhi M. Gomha[1,5]*

[1] Department of Chemistry, Faculty of Science, Islamic University of Madinah, Madinah, 42351, Saudi Arabia

[2] Department of Chemistry, Faculty of Science, Zagazig University, Zagazig 44519, Egypt

[3] Department of Chemistry, College of Science, Princess Nourah bint Abdulrahman University, P.O .BOX 84428, Riyadh 11671, Saudi Arabia

[4] Department of Chemistry, Faculty of Science, Imam Mohammad Ibn Saud Islamic University (IMSIU), Riyadh 11623, Saudi Arabia

[5] Department of Chemistry, Faculty of Science, Cairo University, Cairo 12613, Egypt

Correspondence: smgomha@iu.edu.sa

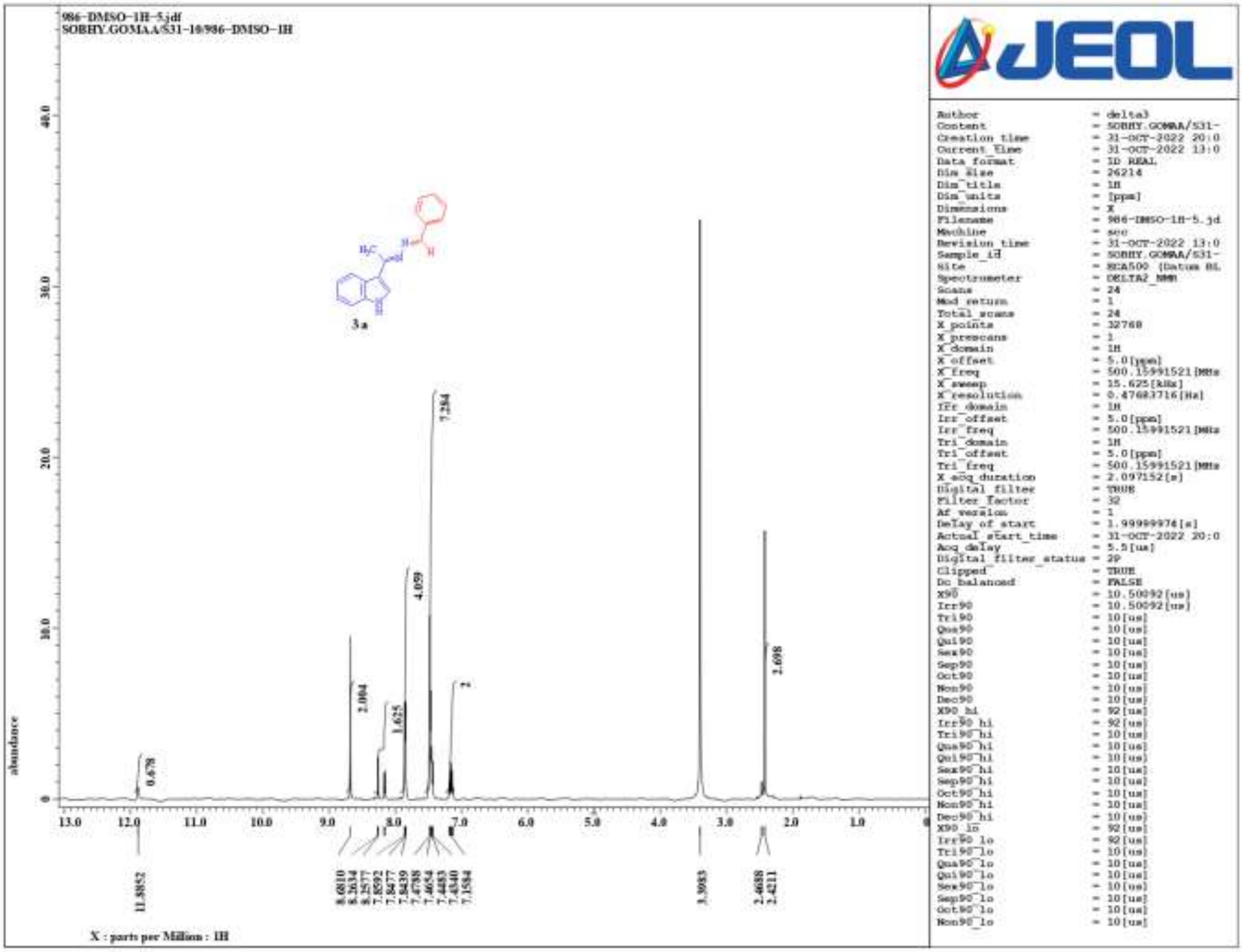

^{1}H-NMR spectra of compound **3a**

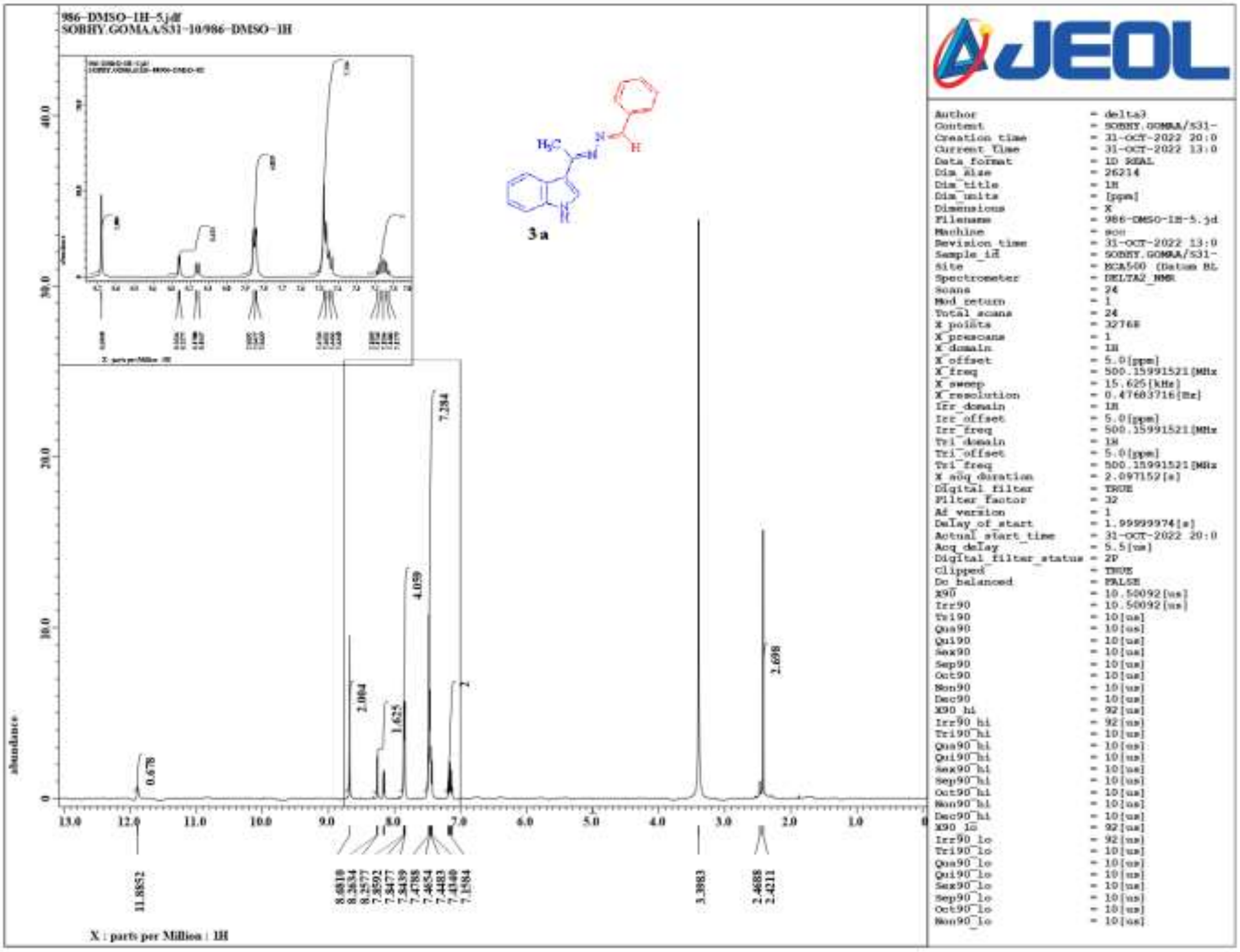

^{1}H-NMR spectra of compound **3a** (magnified aromatic region)

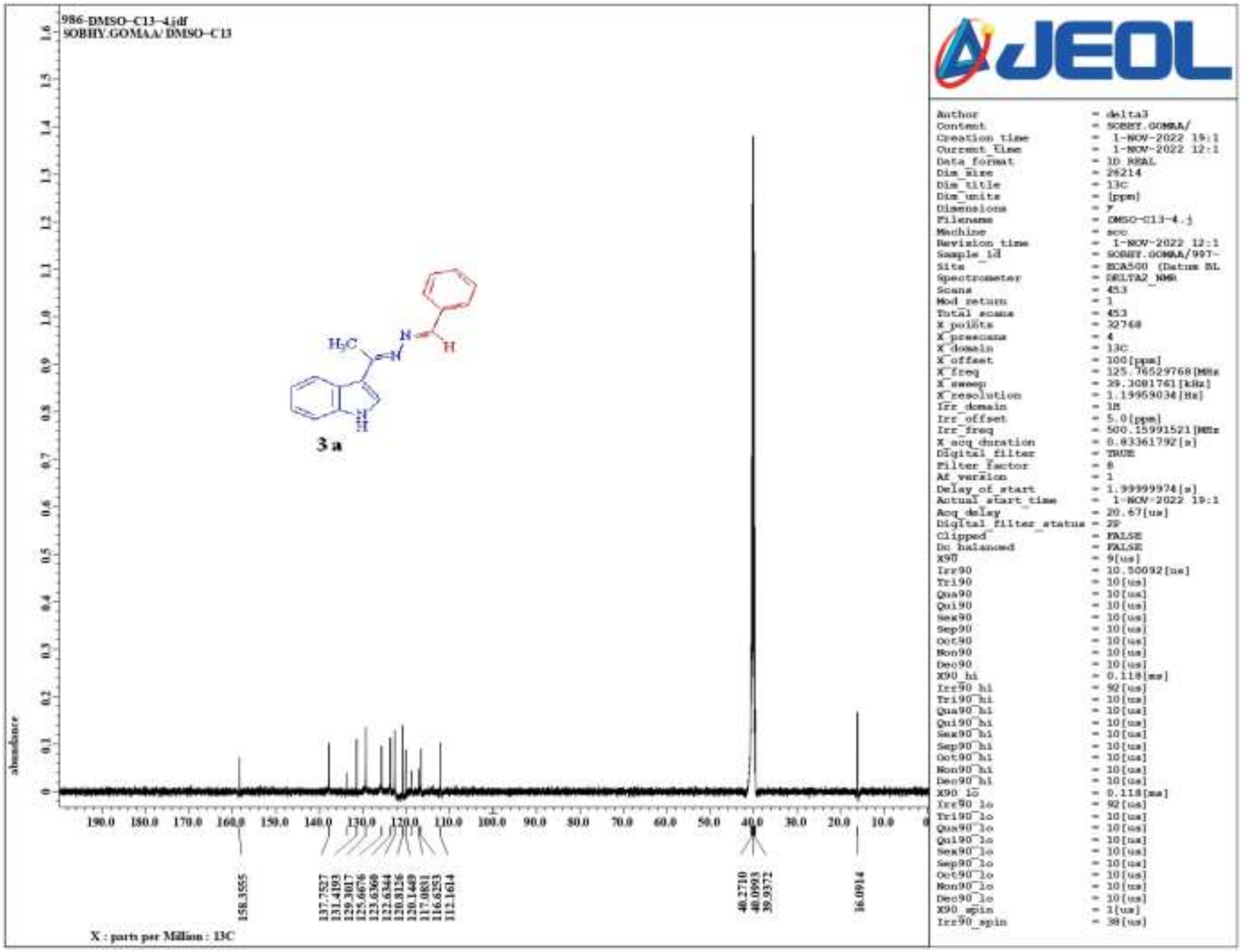

^{13}C-NMR spectra of compound **3a**

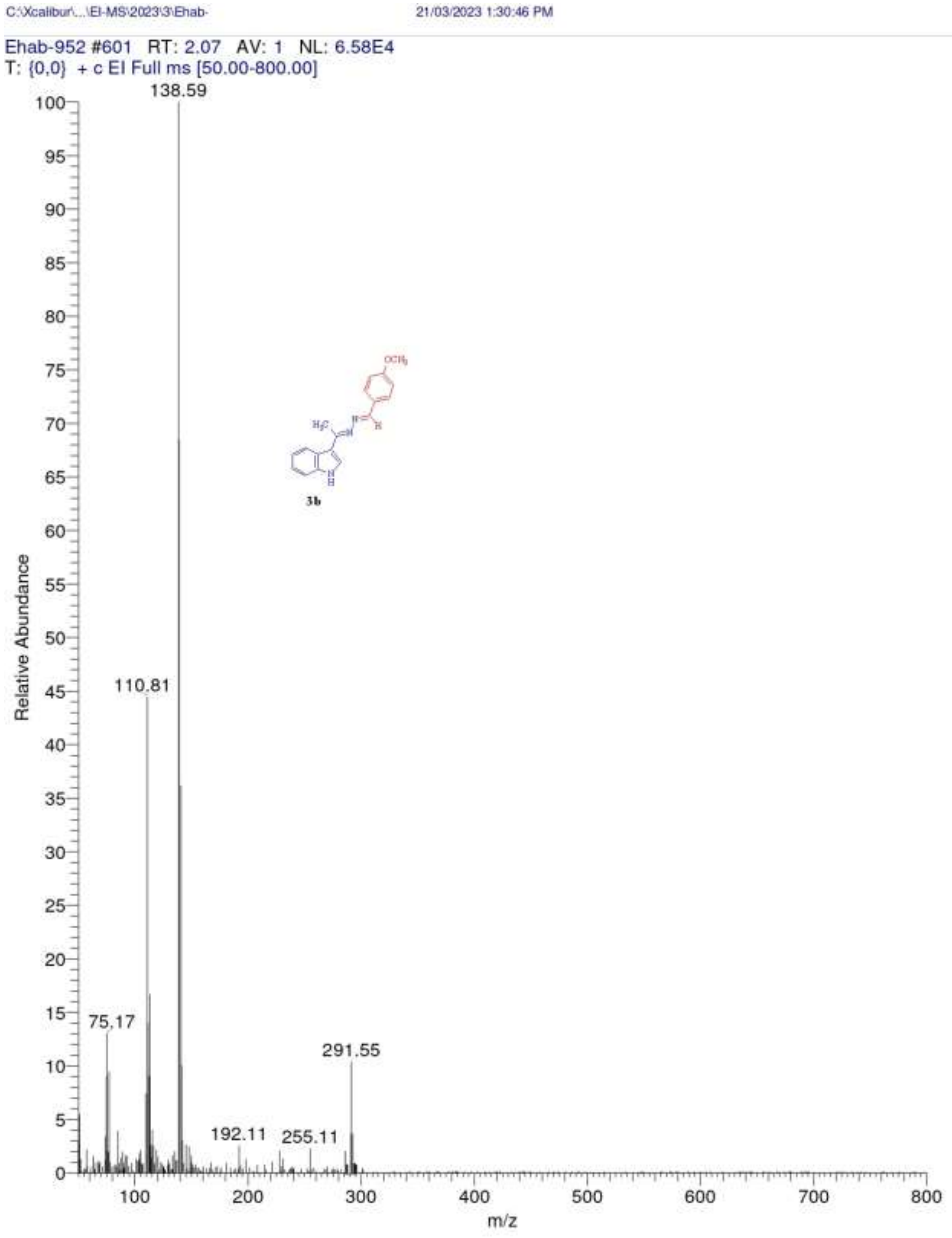

Mass spectra of compound **3b**

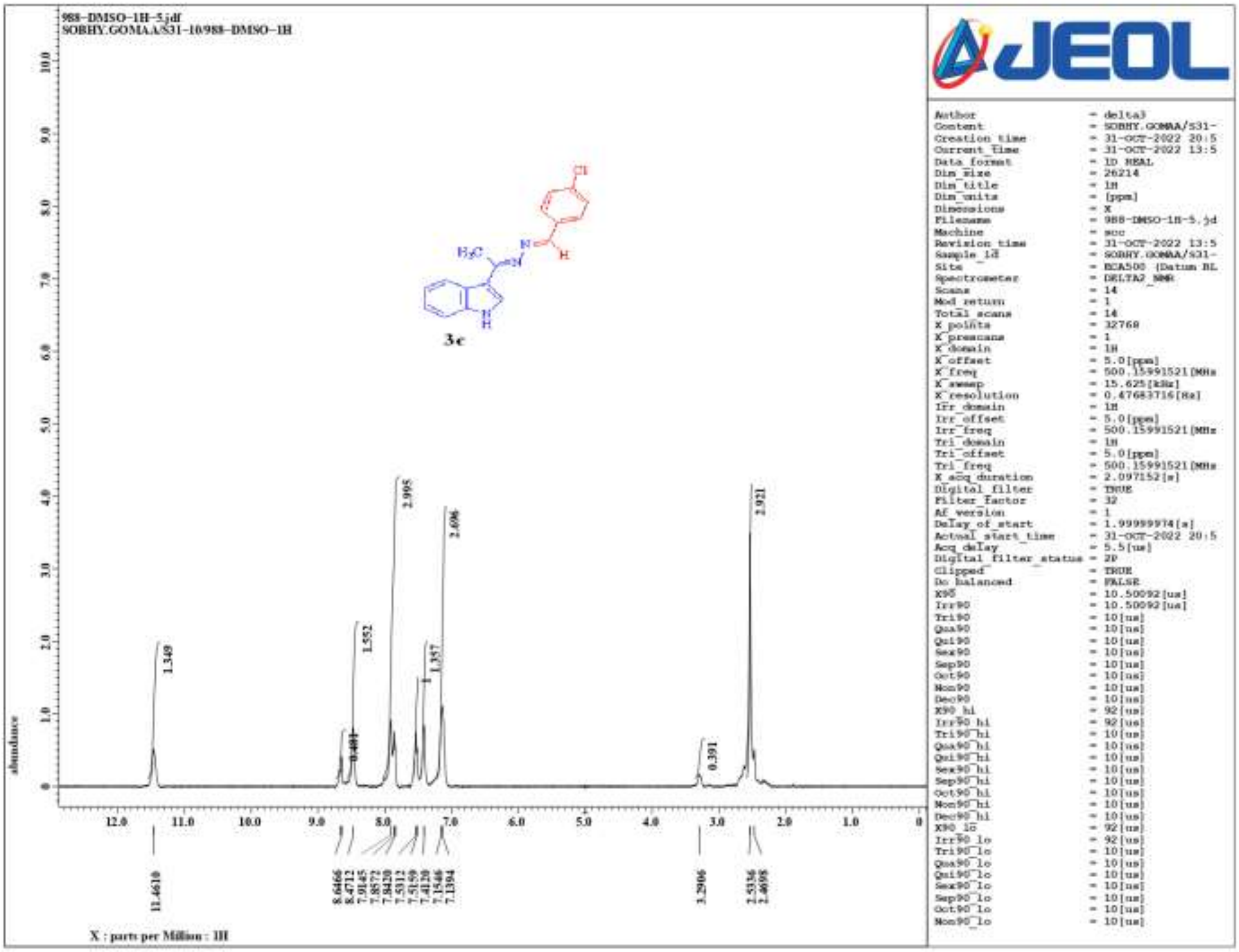

^{1}H-NMR spectra of compound **3c**

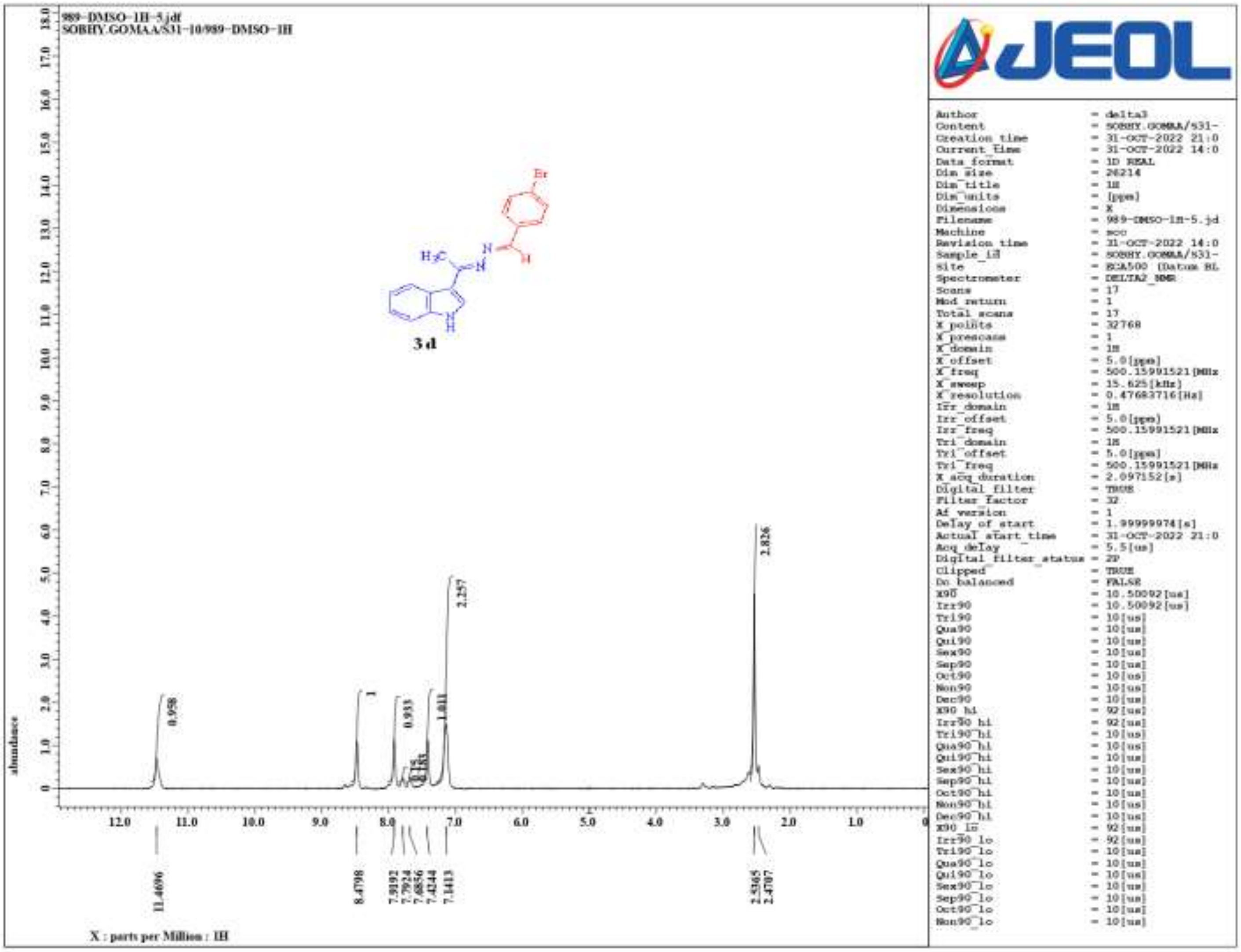

^{1}H-NMR spectra of compound **3d**

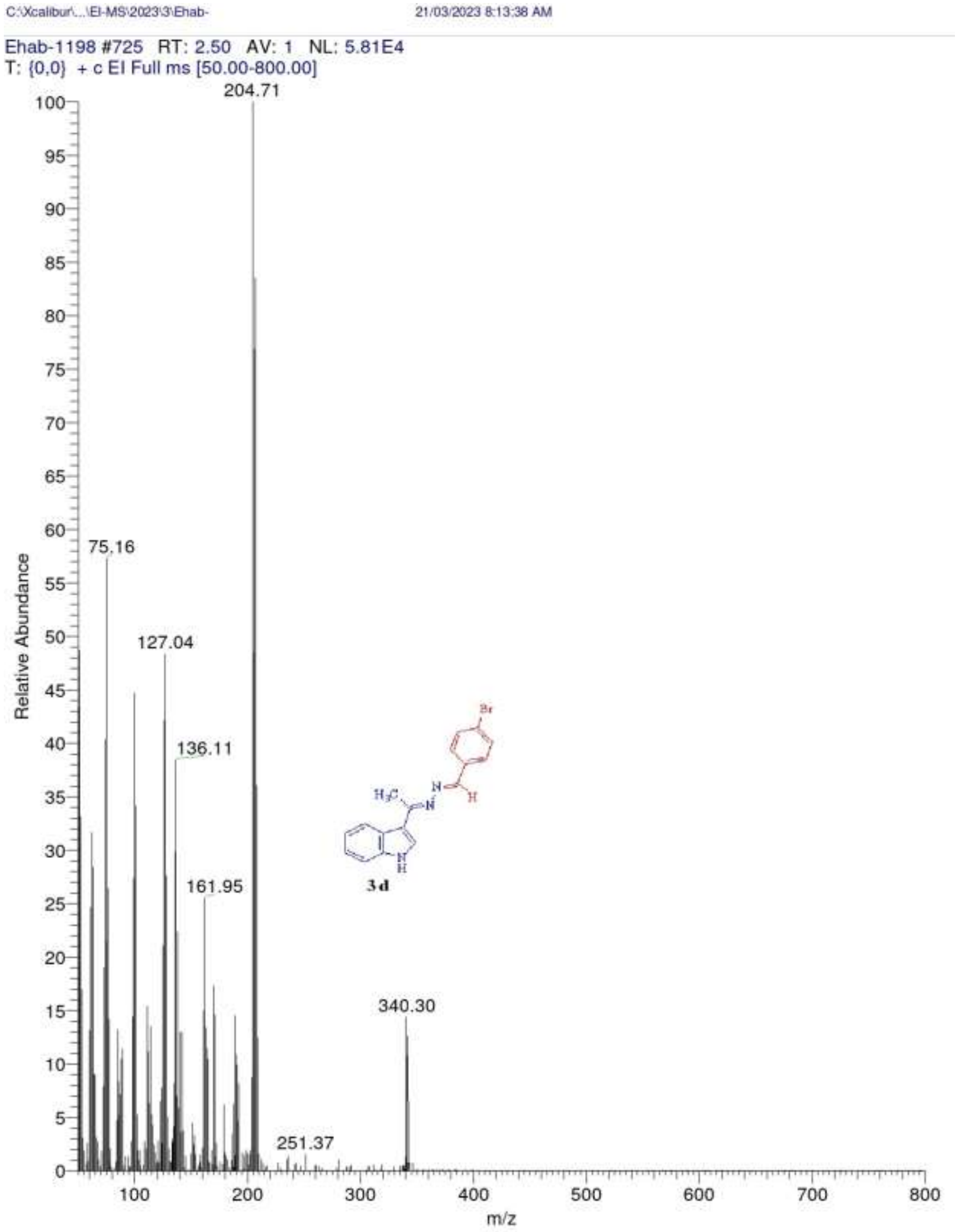

Mass spectra of compound **3d**

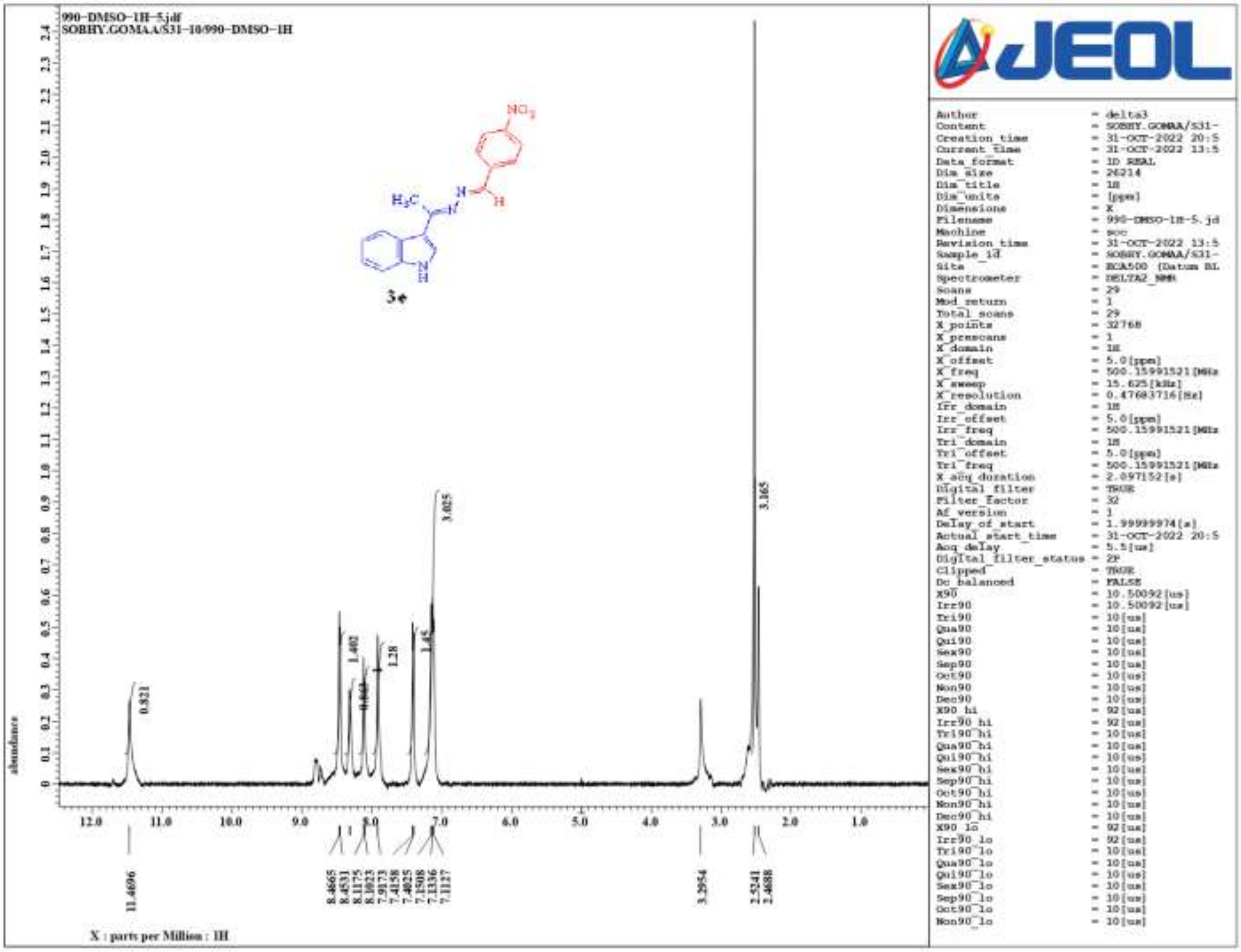

^{1}H-NMR spectra of compound **3e**

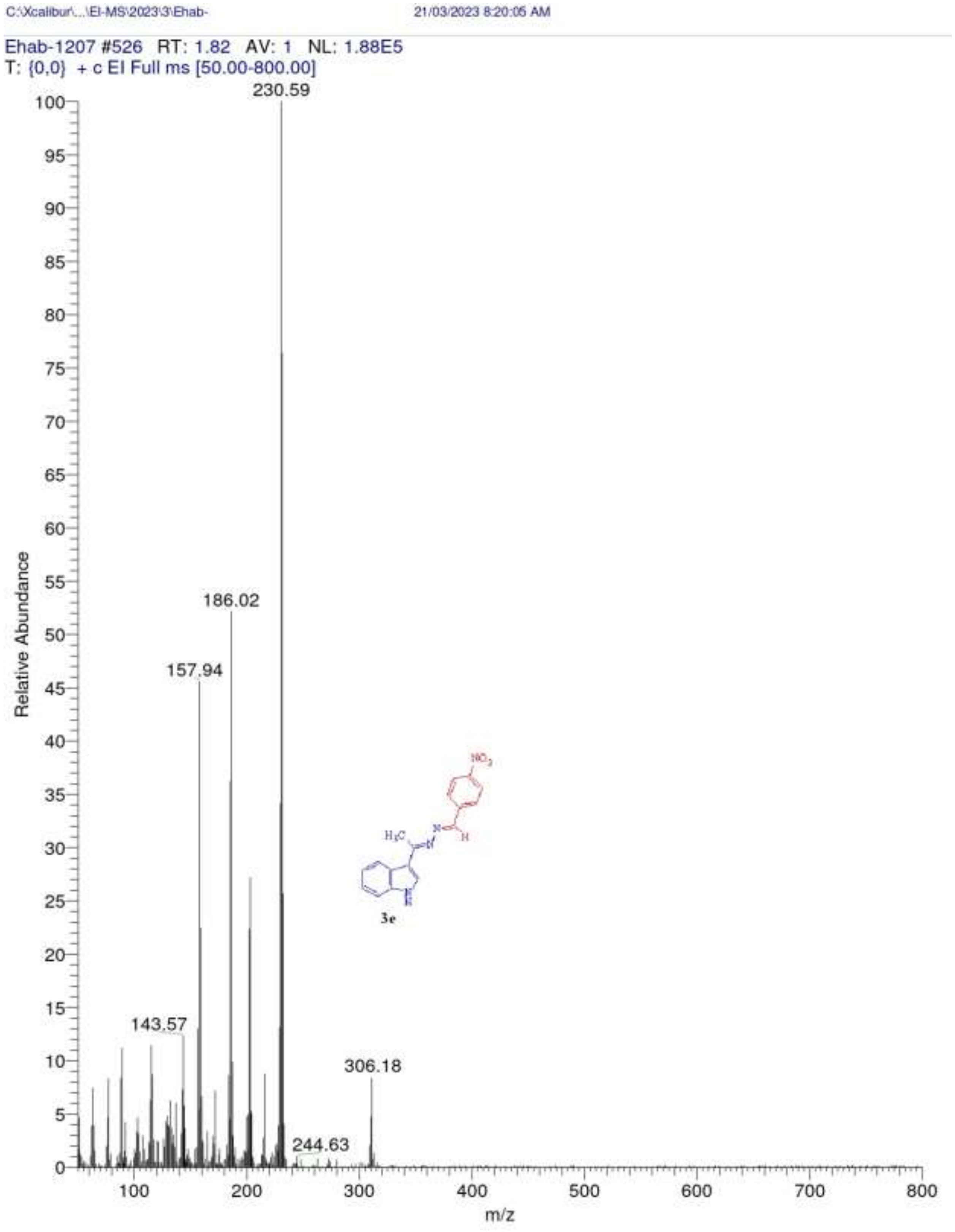

Mass spectra of compound **3e**

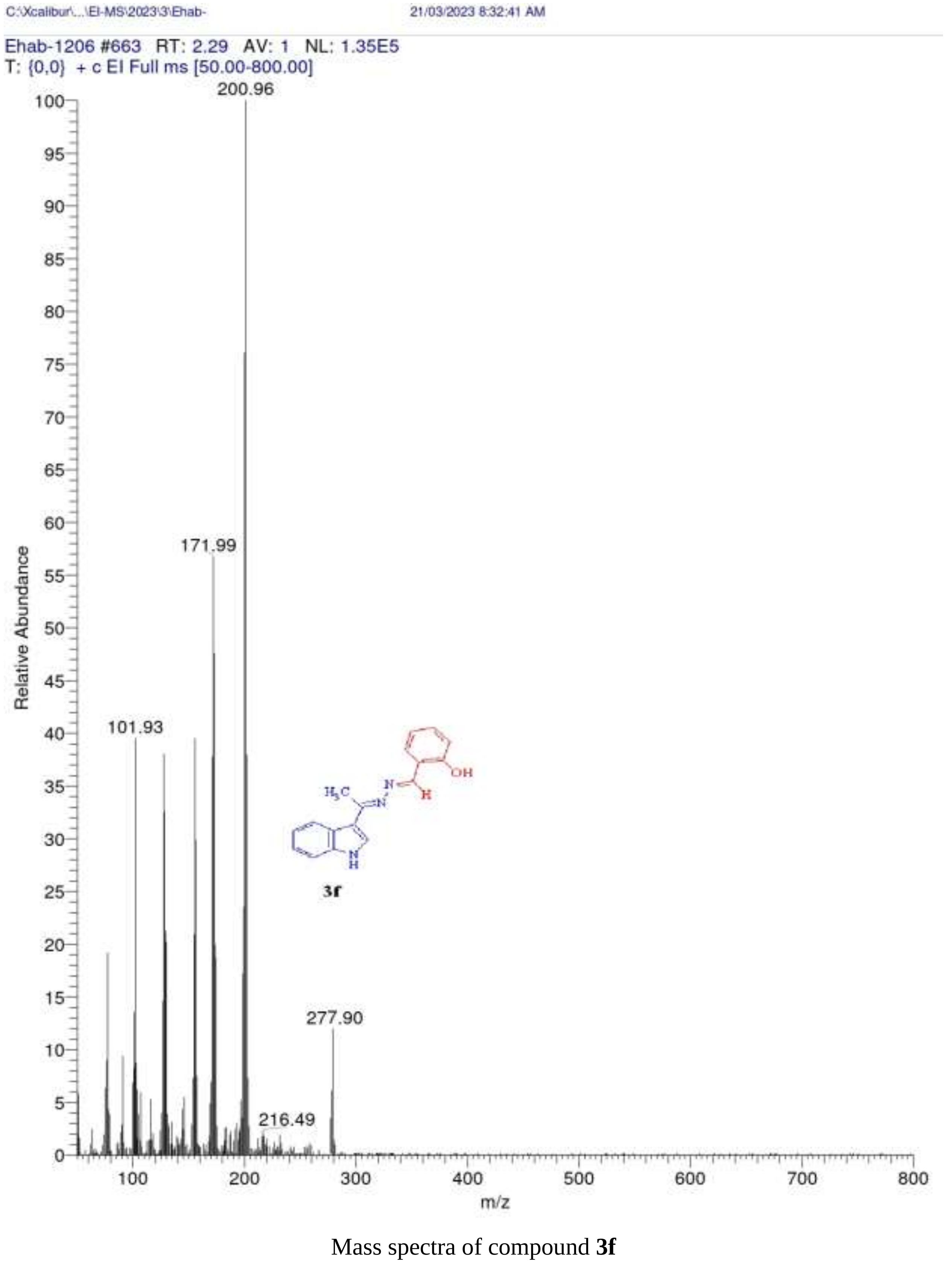

Mass spectra of compound **3f**

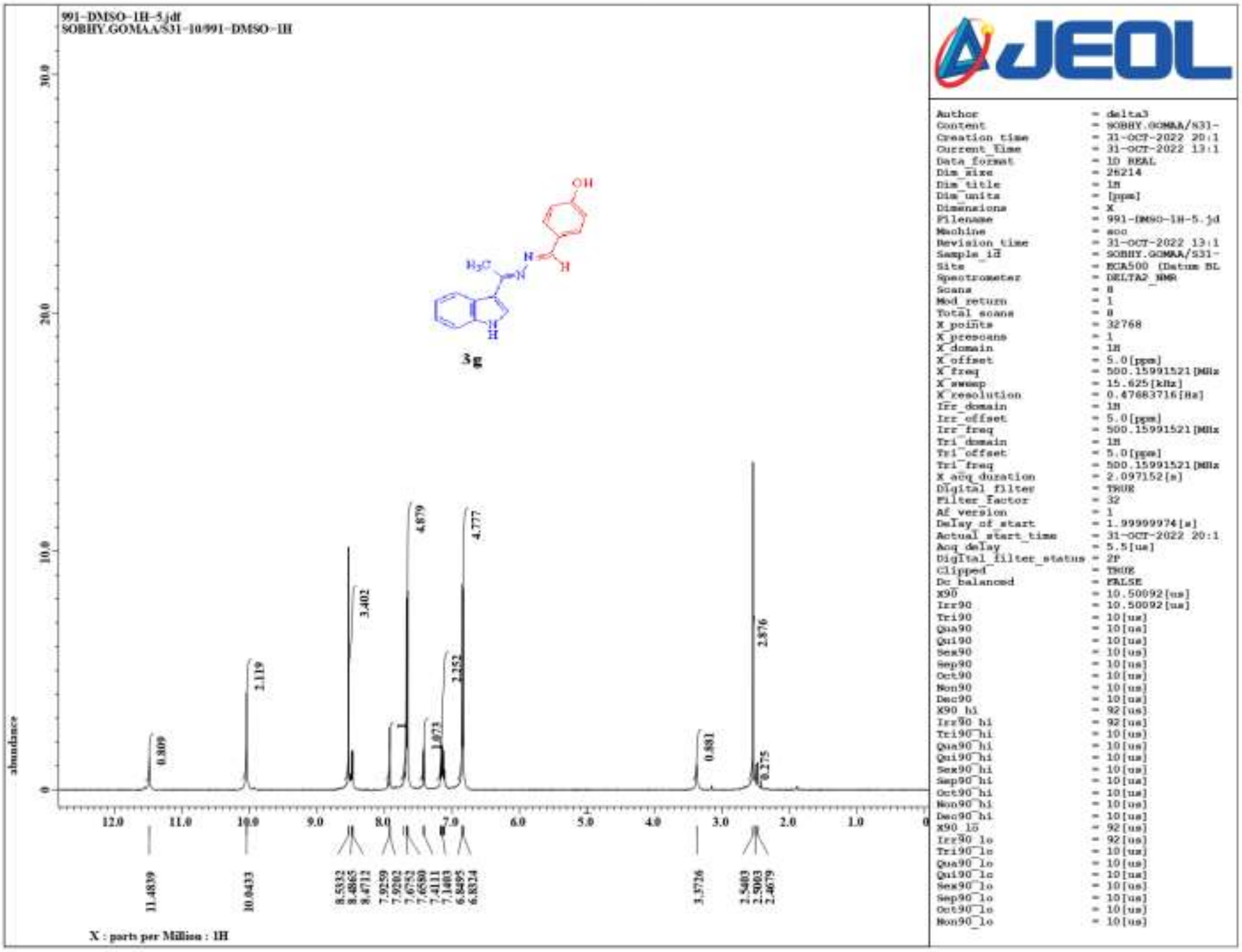

^{1}H-NMR spectra of compound **3g**

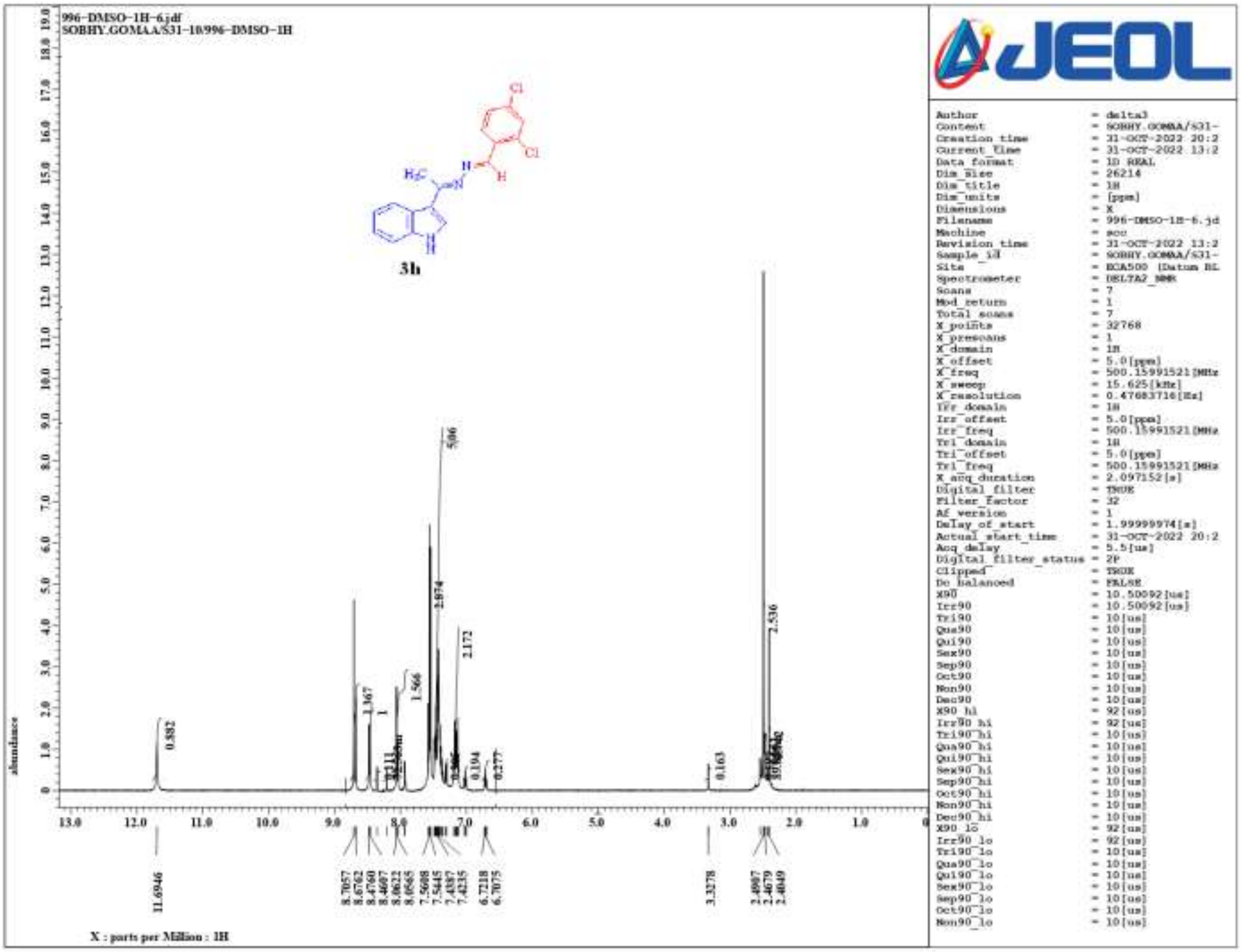

^{1}H-NMR spectra of compound **3h**

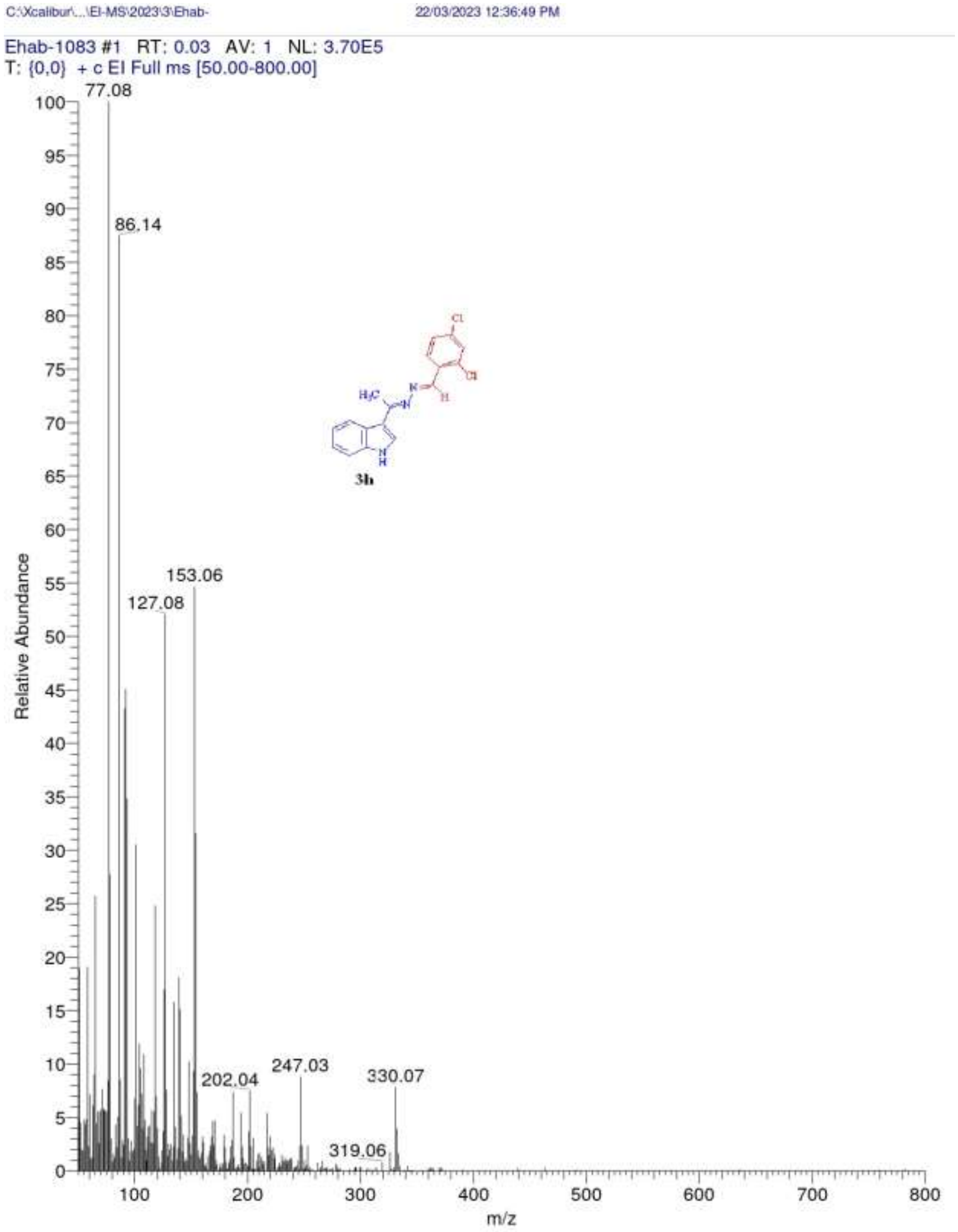

Mass spectra of compound **3h**

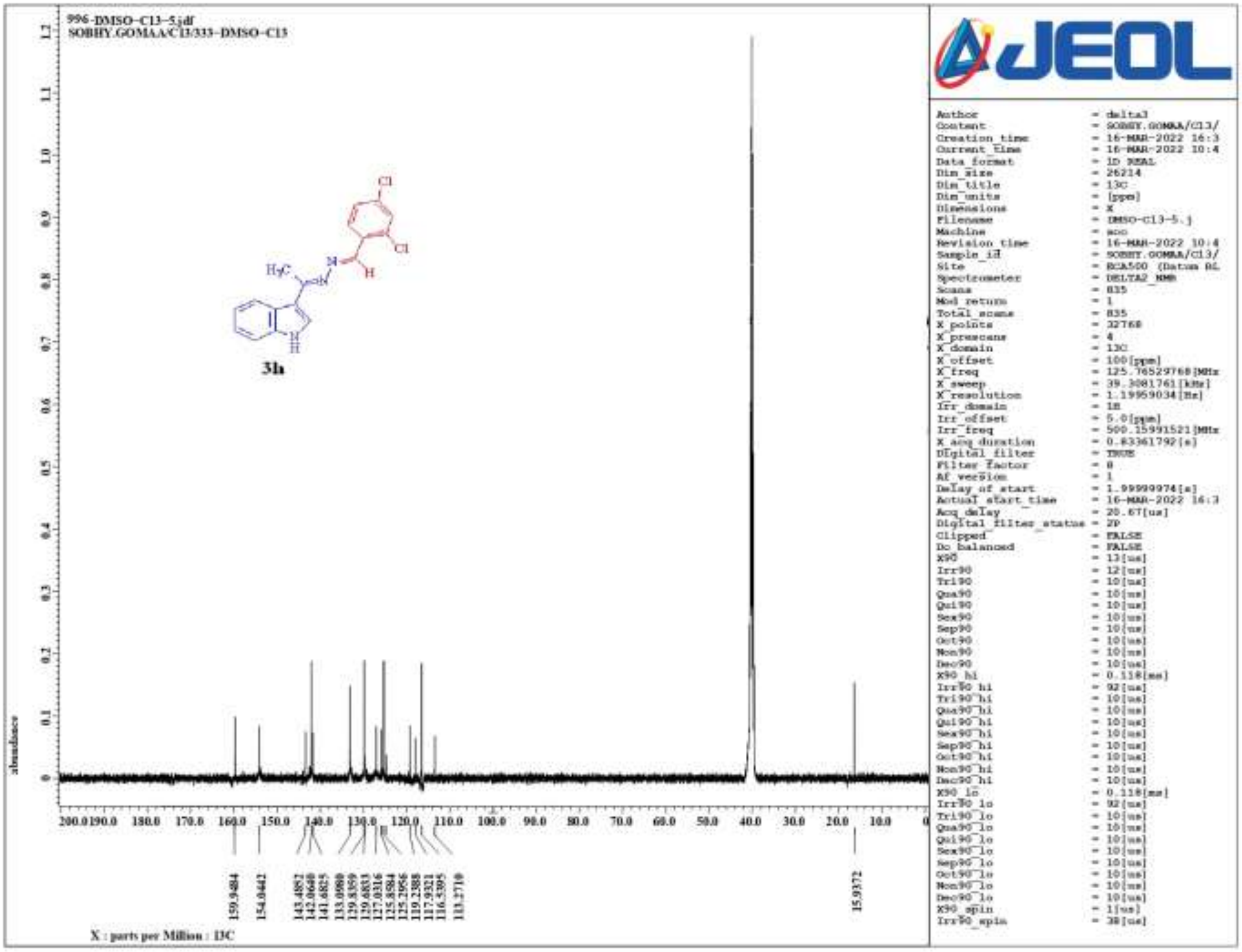

^{13}C-NMR spectra of compound **3h**

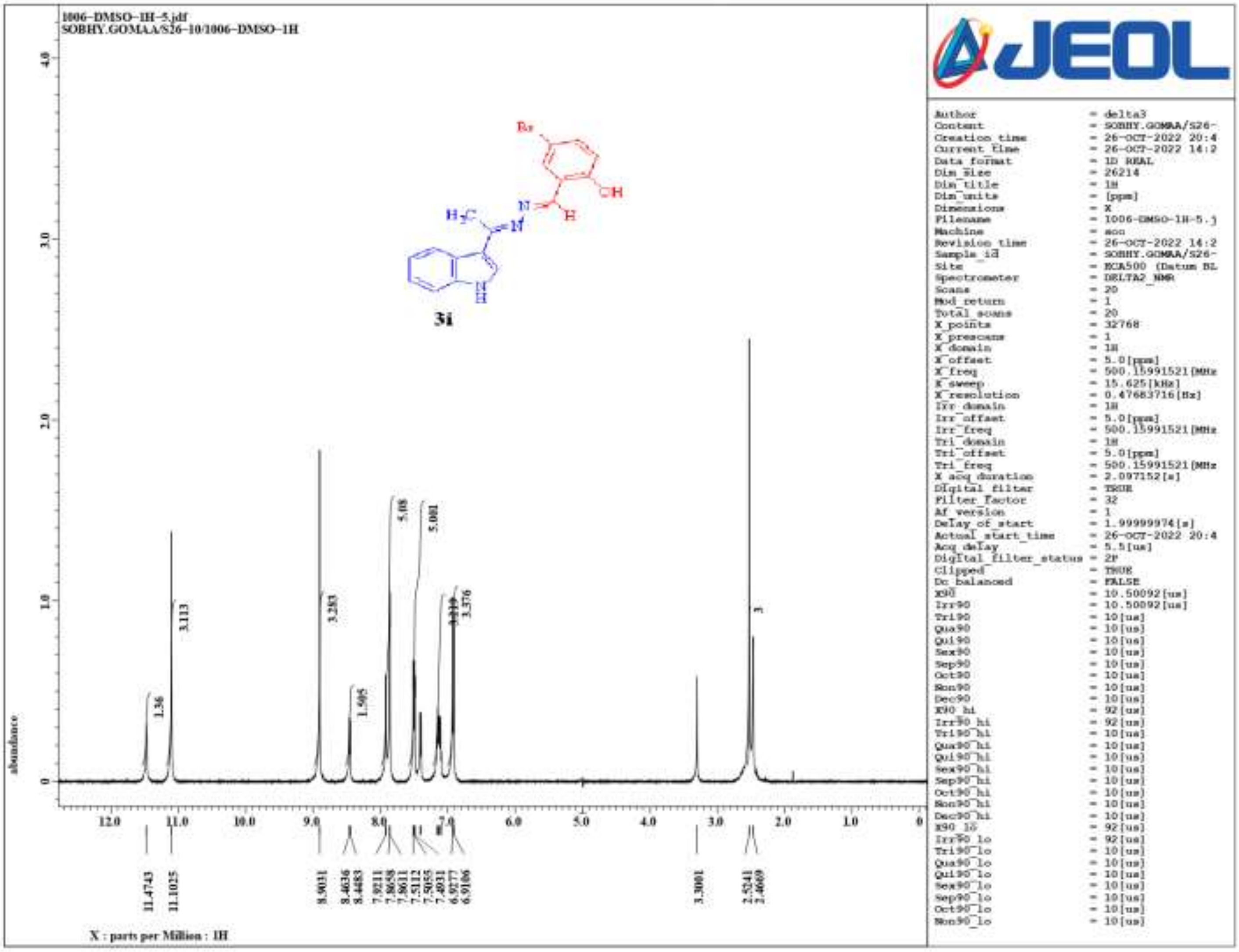

^{1}H-NMR spectra of compound **3i**

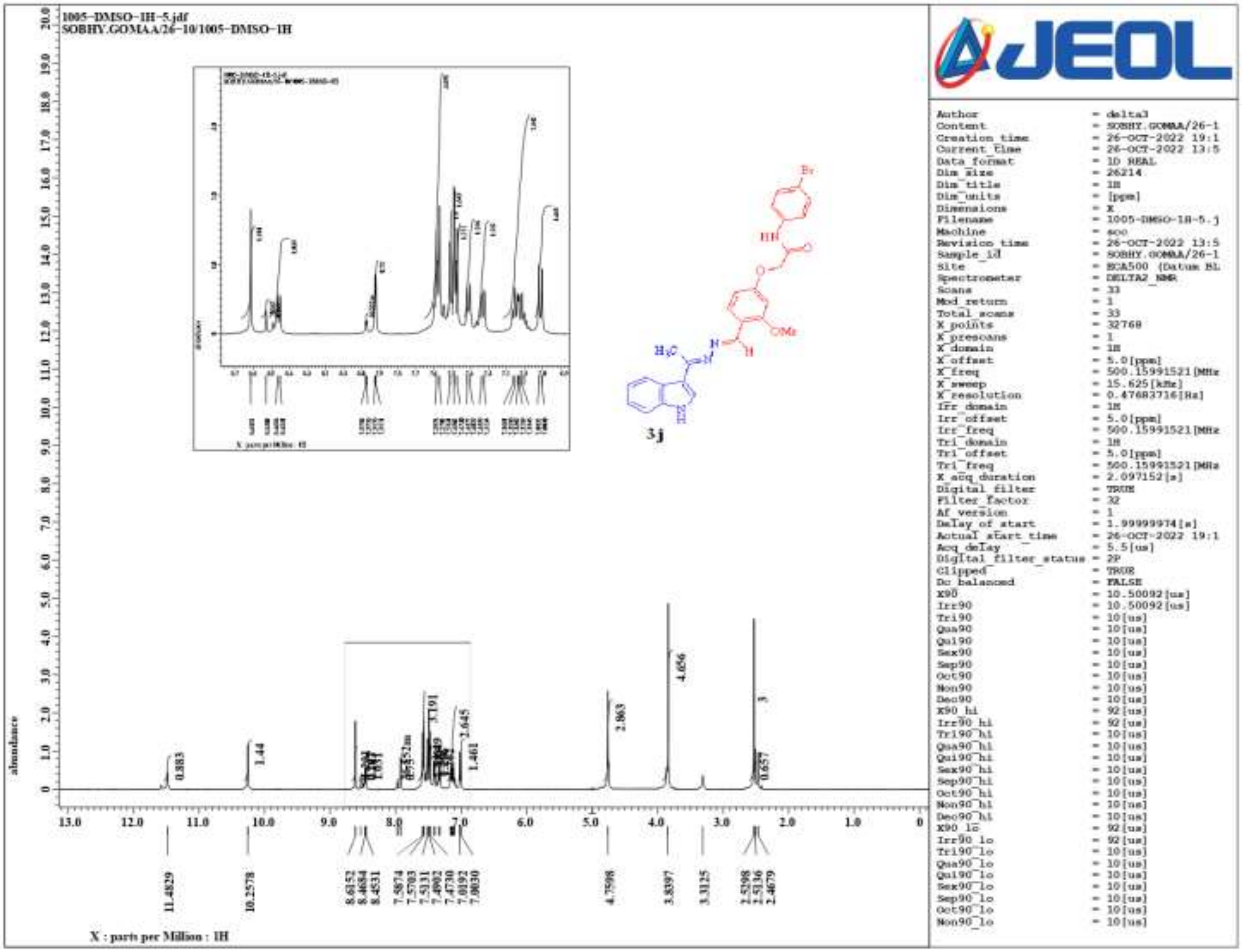

^{1}H-NMR spectra of compound **3j**

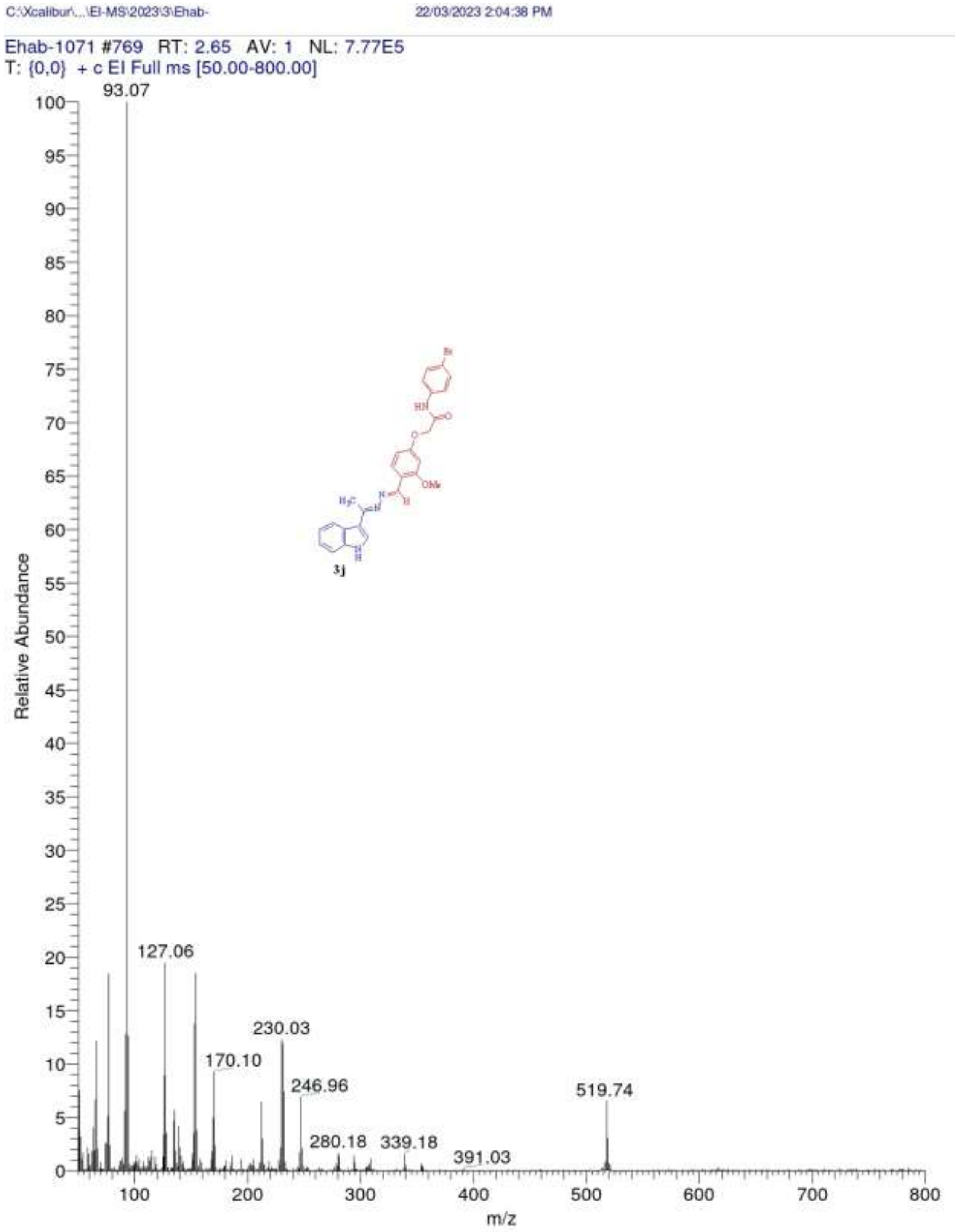

Mass spectra of compound **3j**

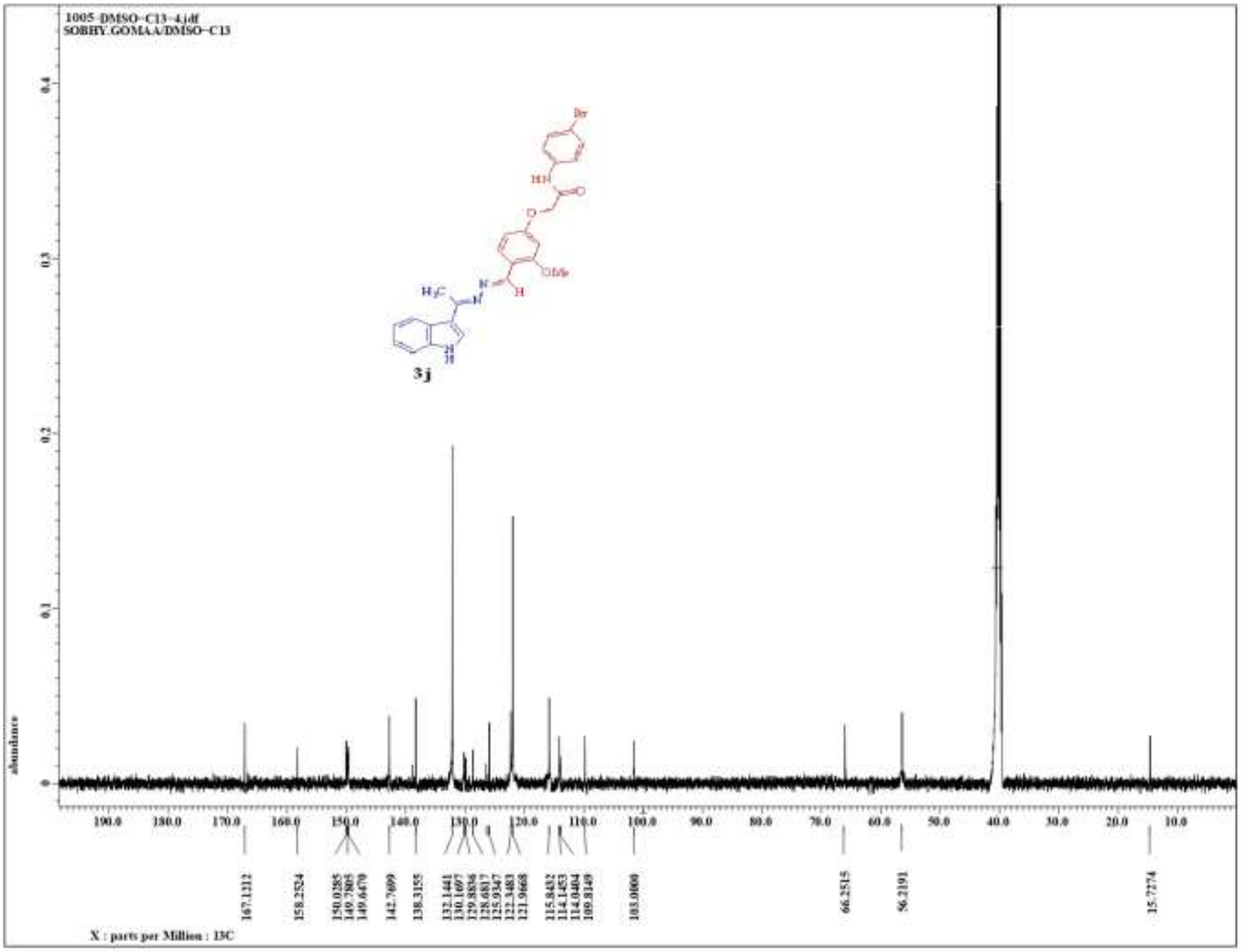

^{13}C-NMR spectra of compound **3j**

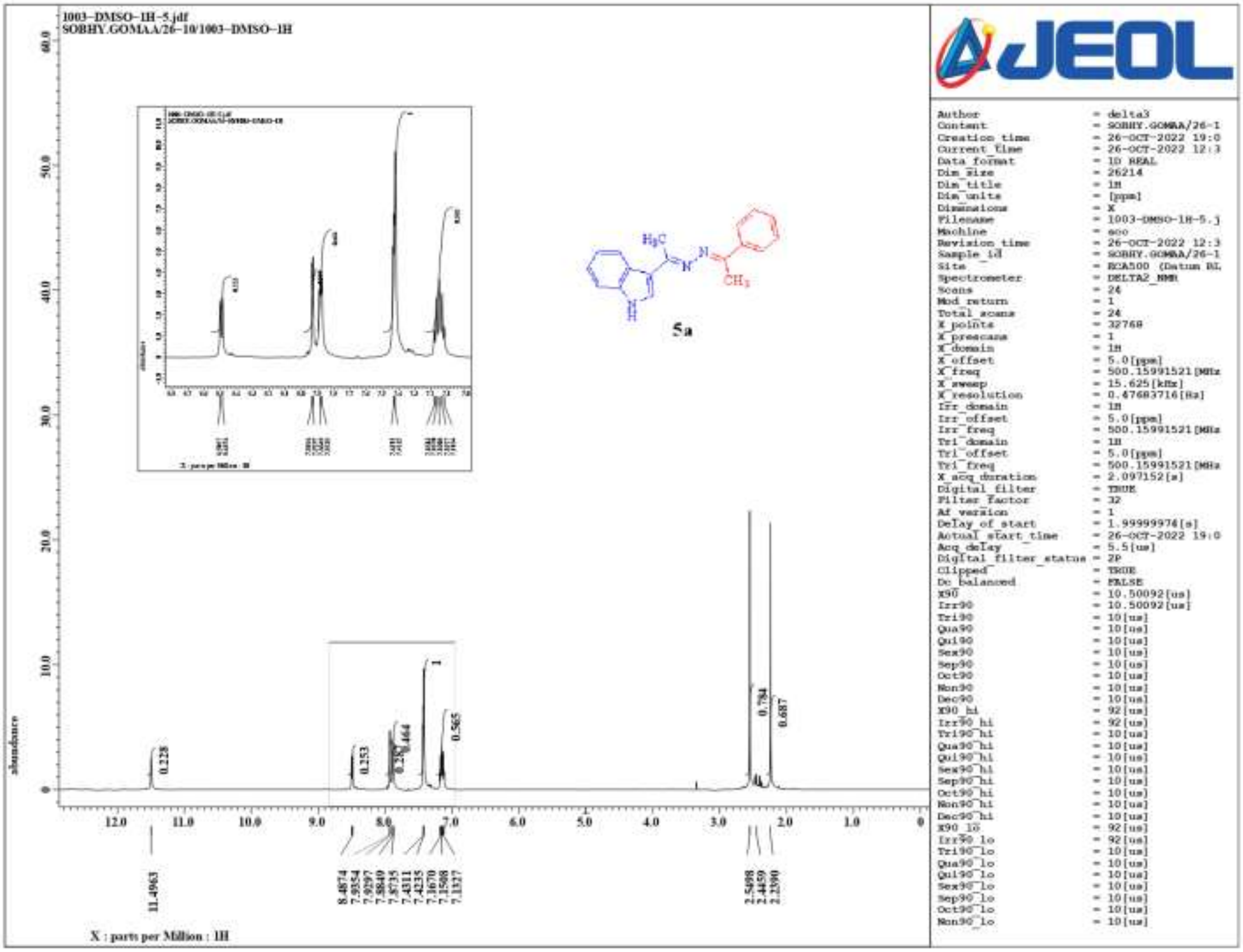

^{1}H-NMR spectra of compound **5a**

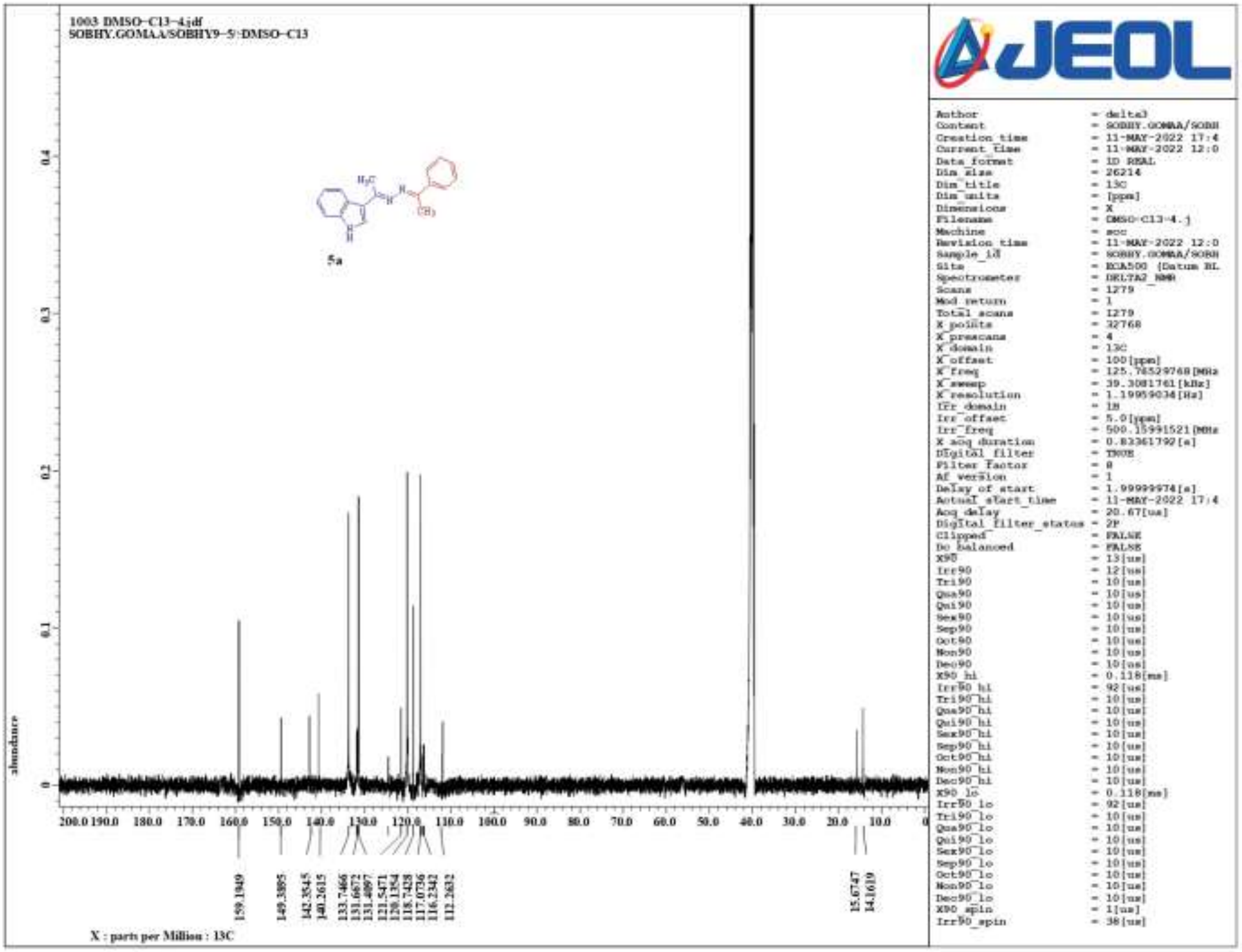

^{13}C-NMR spectra of compound **5a**

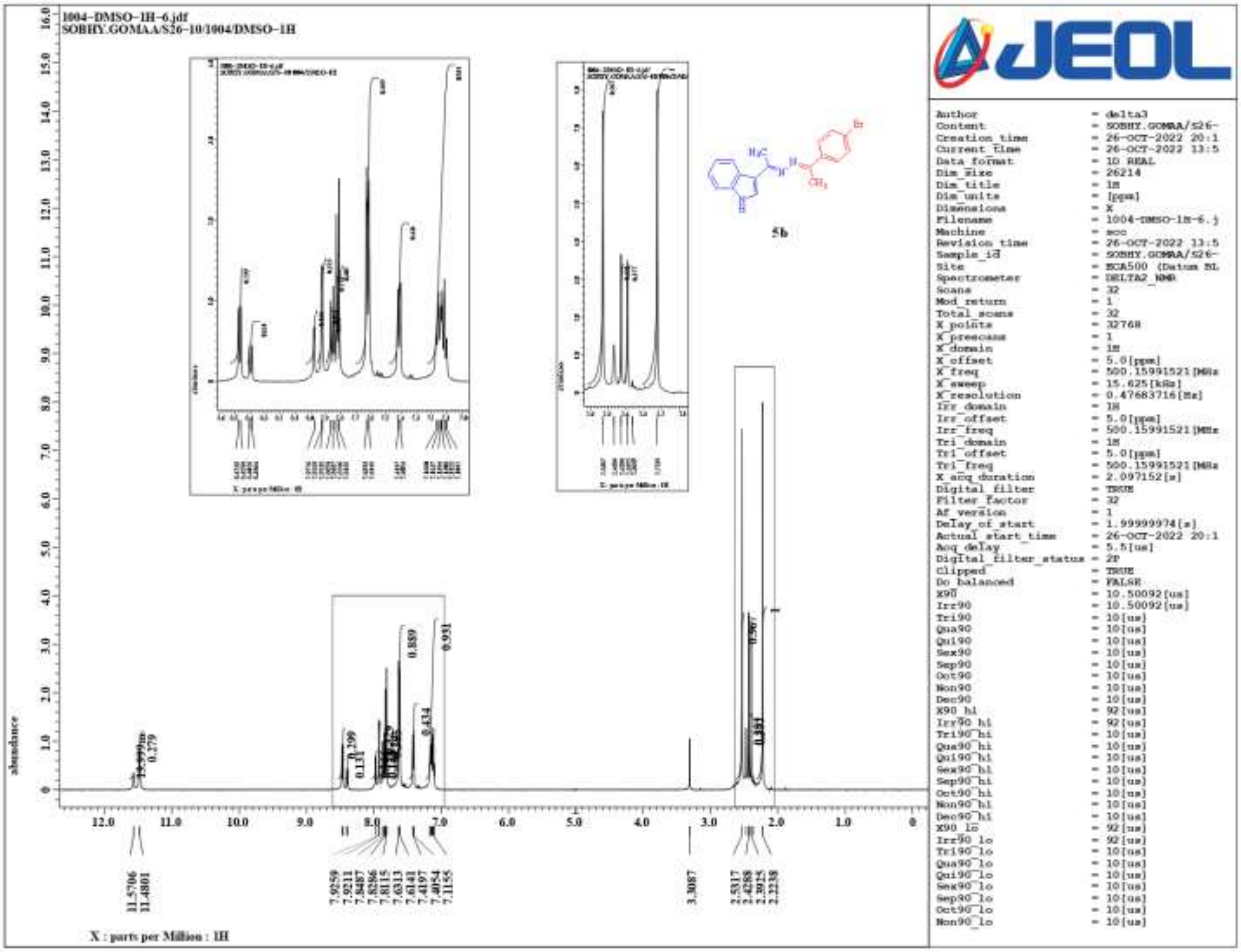

^{1}H-NMR spectra of compound **5b**

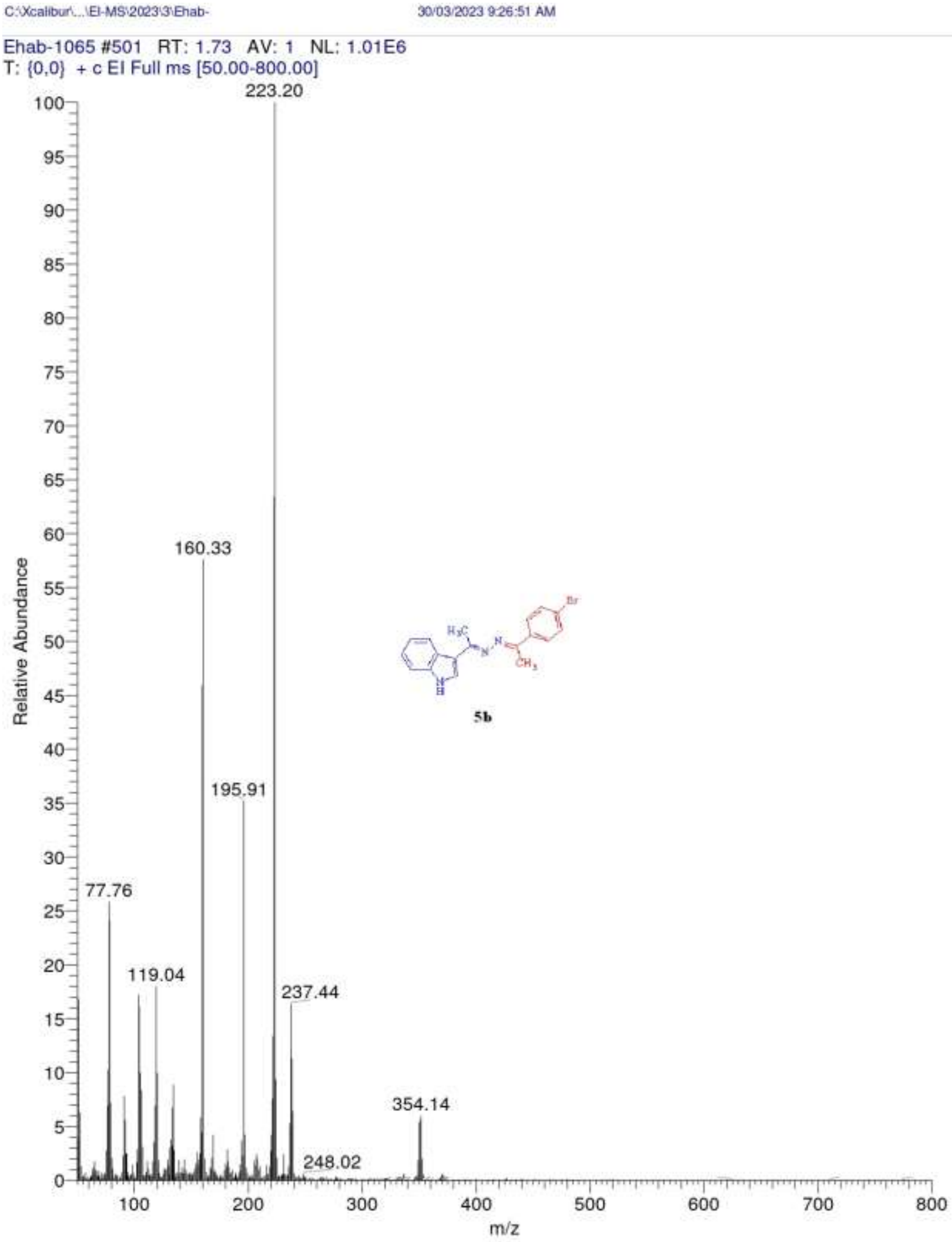

Mass spectra of compound **5b**

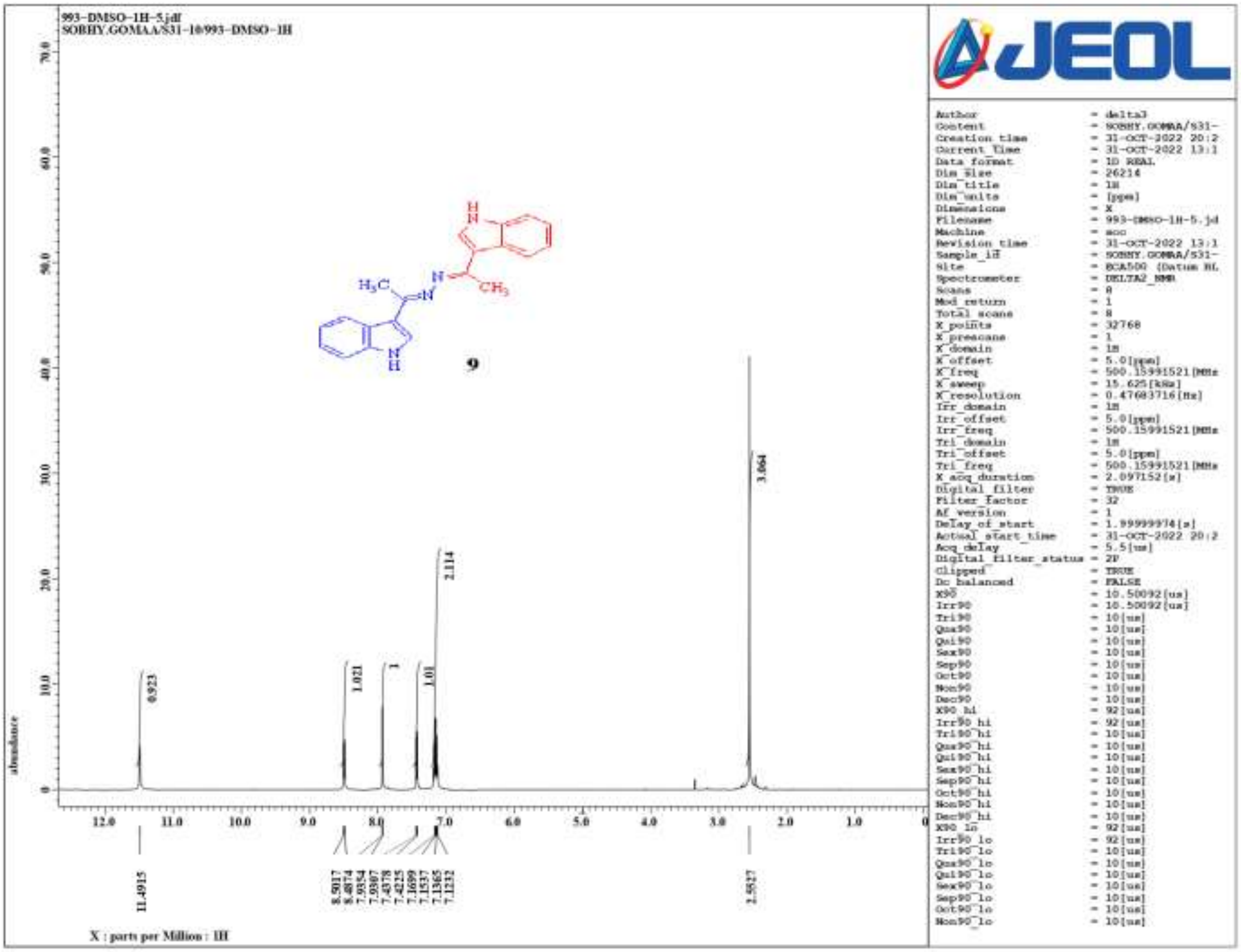

^{1}H-NMR spectra of compound **9**

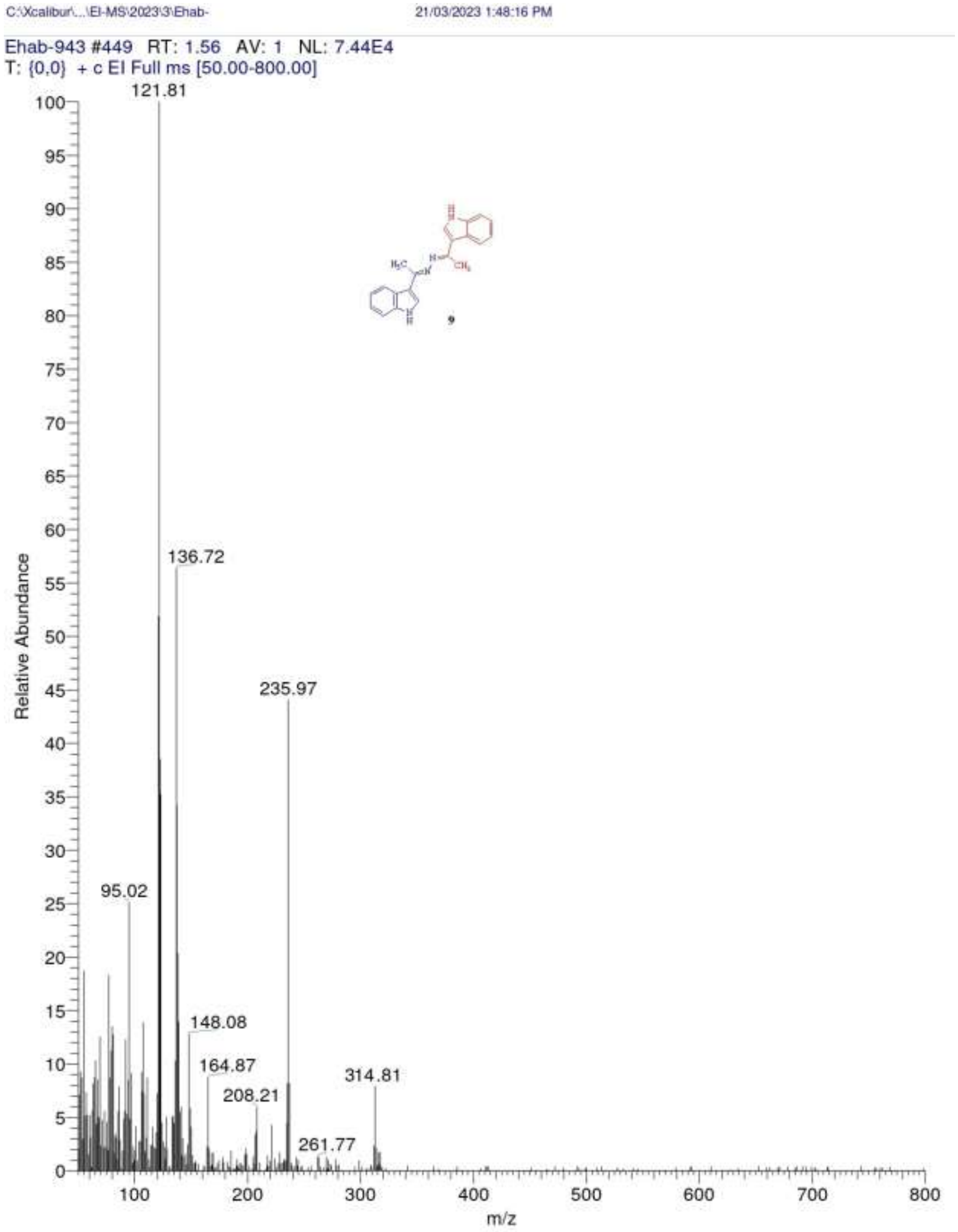

Mass spectra of compound **9**

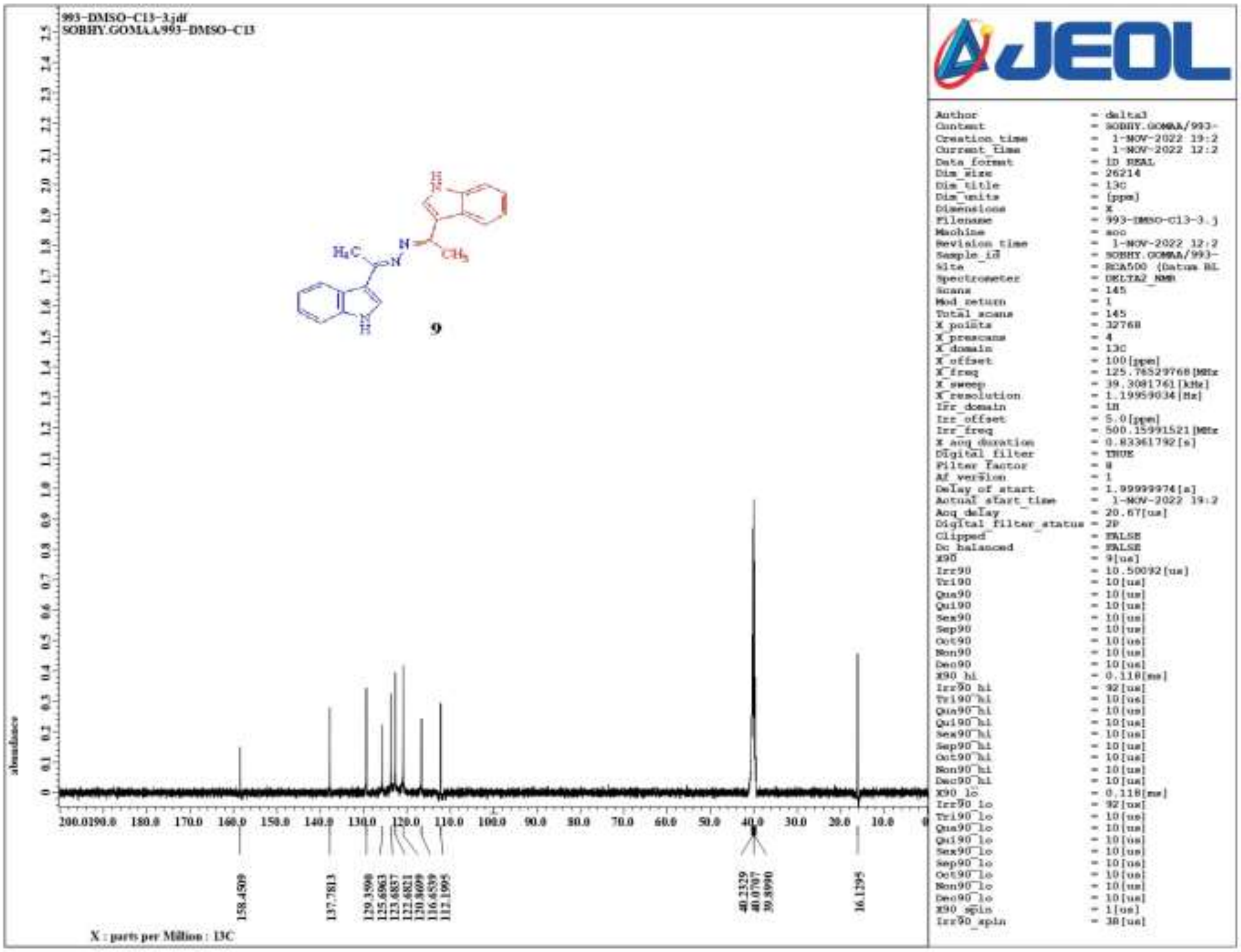

^{13}C-NMR spectra of compound **9**

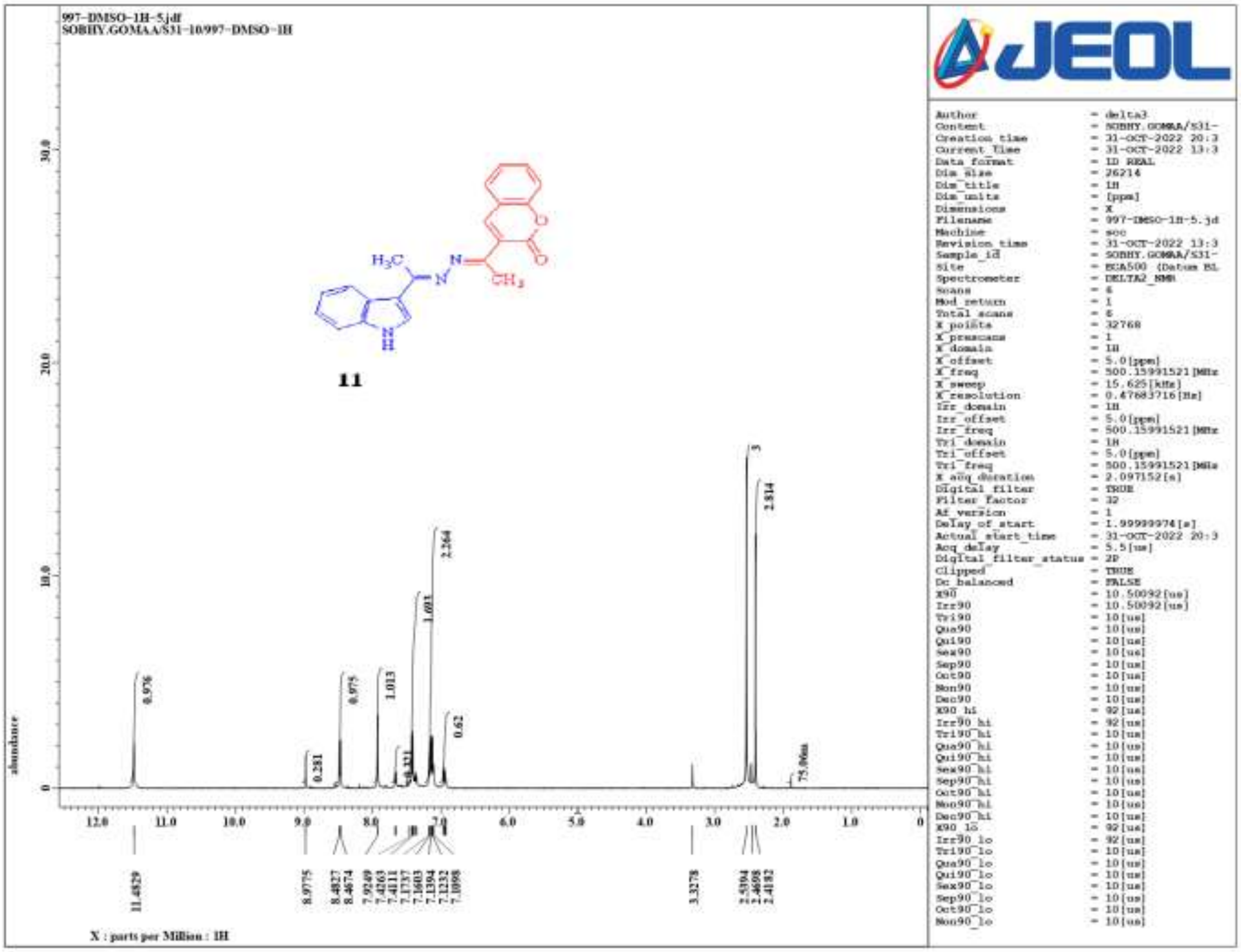

^{1}H-NMR spectra of compound **11**

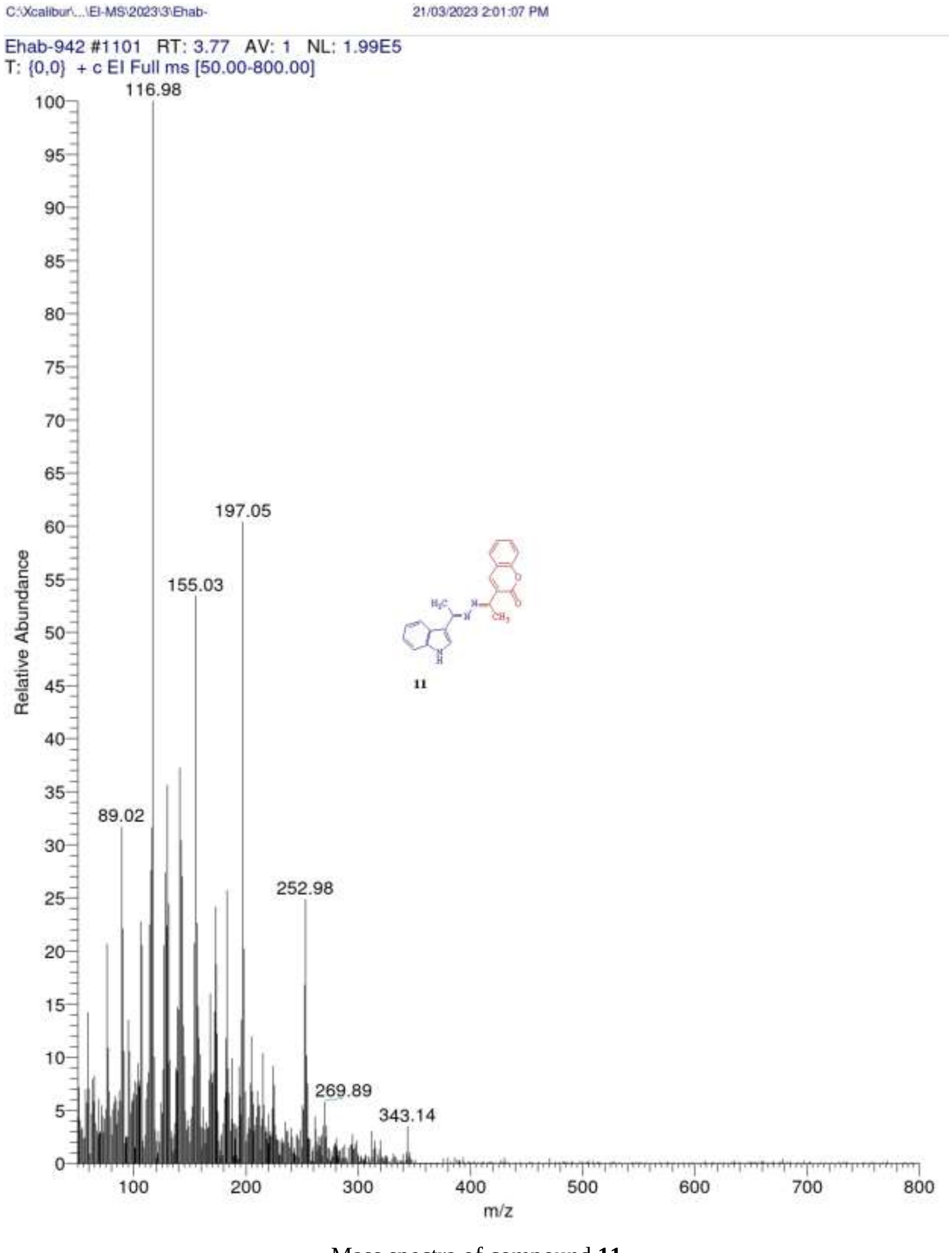

Mass spectra of compound **11**

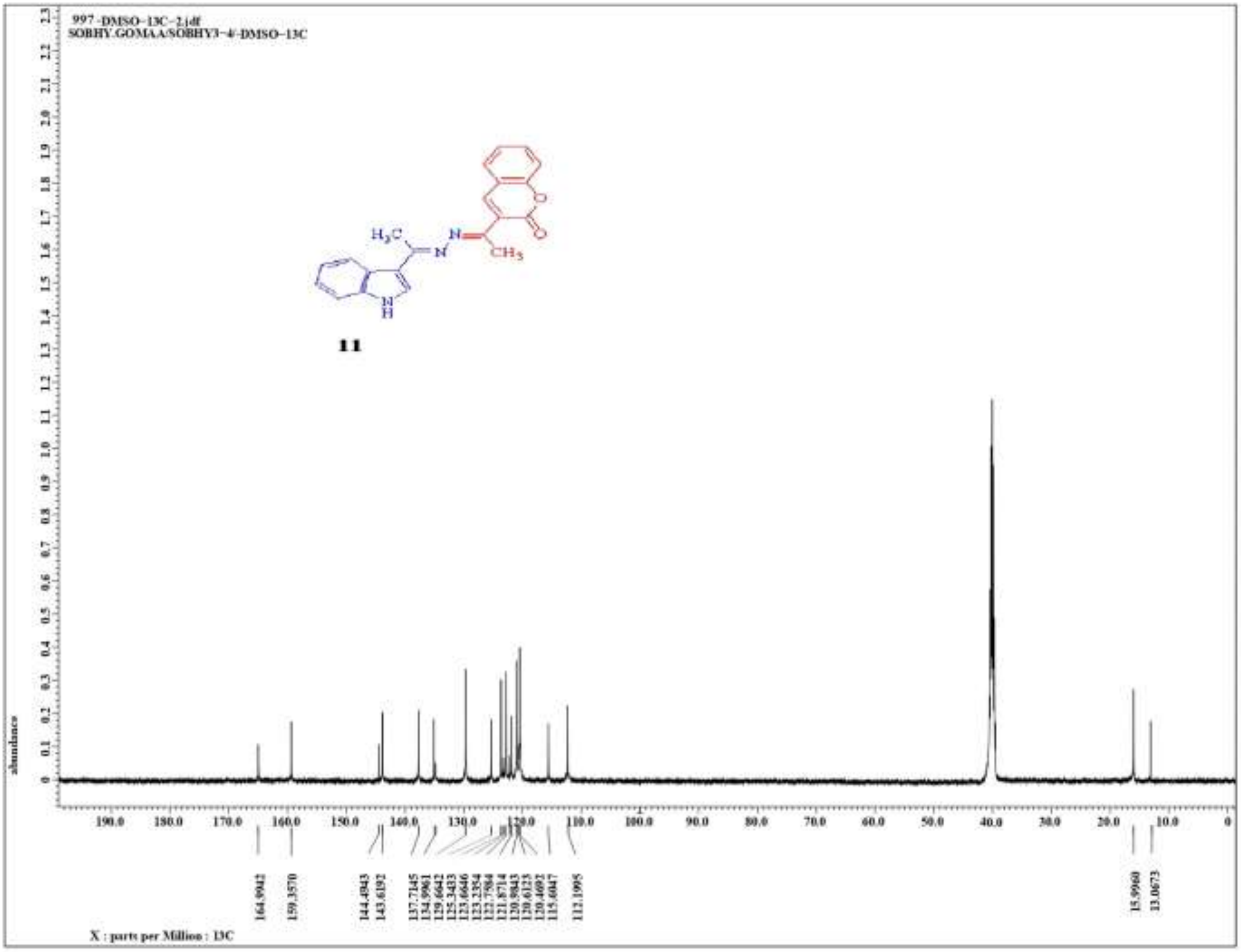

^{13}C-NMR spectra of compound **11**

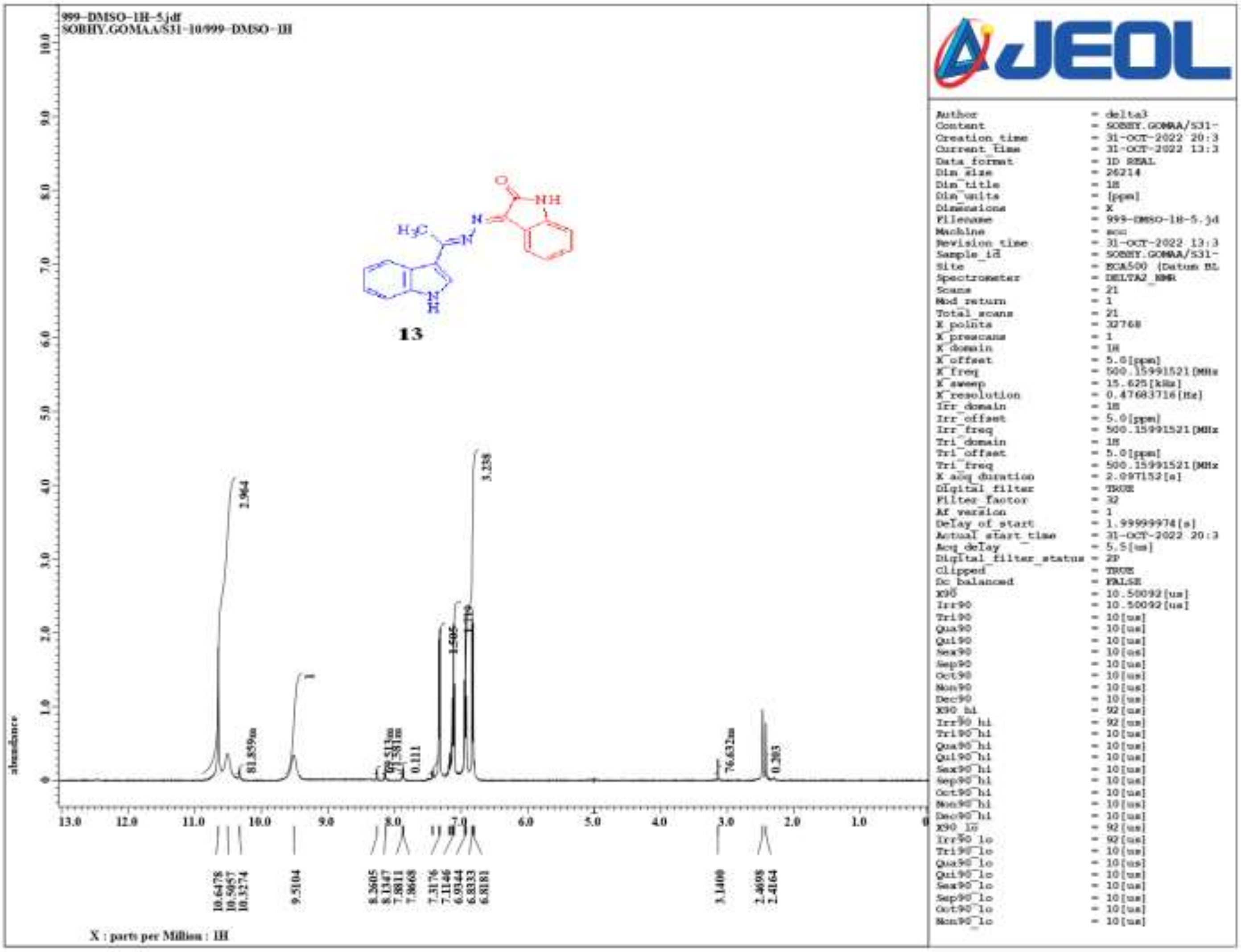

^{1}H-NMR spectra of compound **13**

Compd.	3D Ligand interaction	2D Ligand interaction
3a	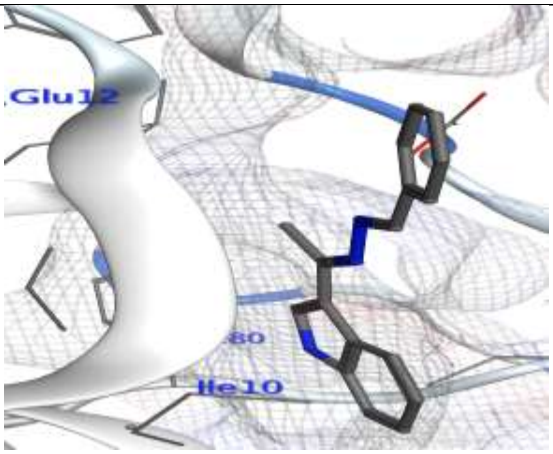	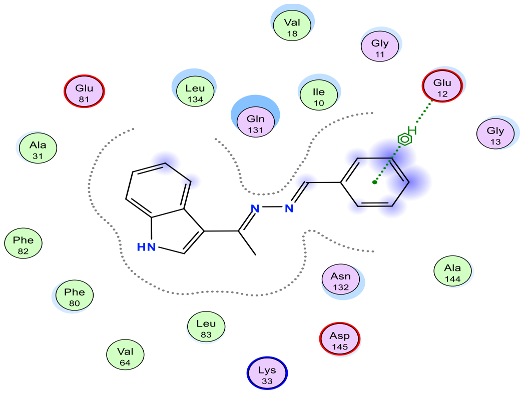
3b	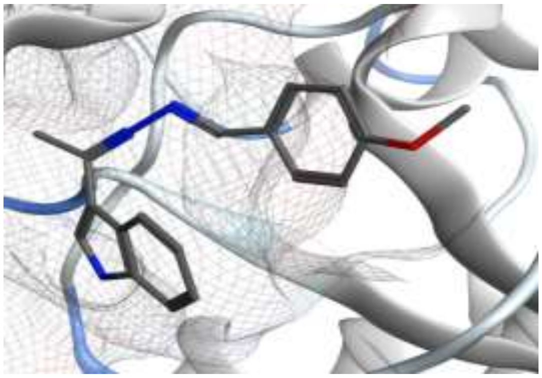	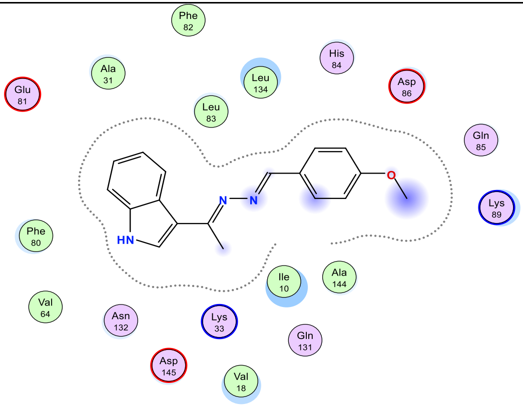
3c	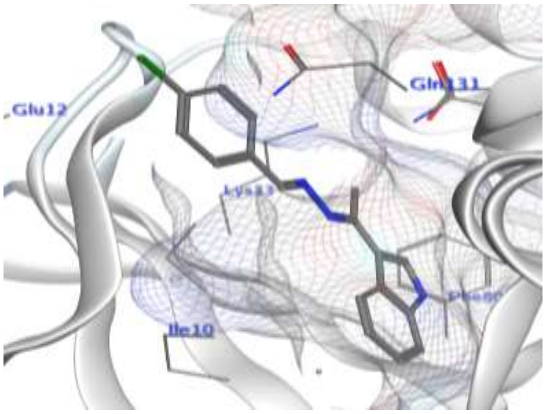	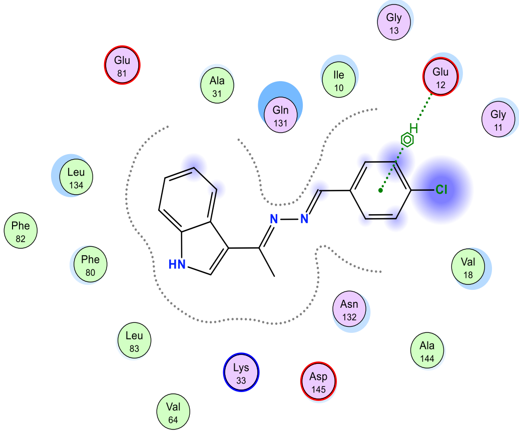

**3d**	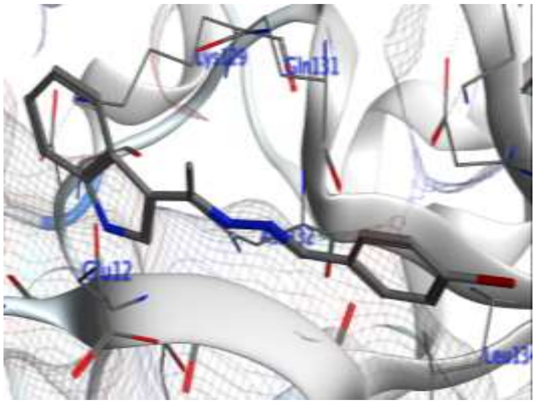	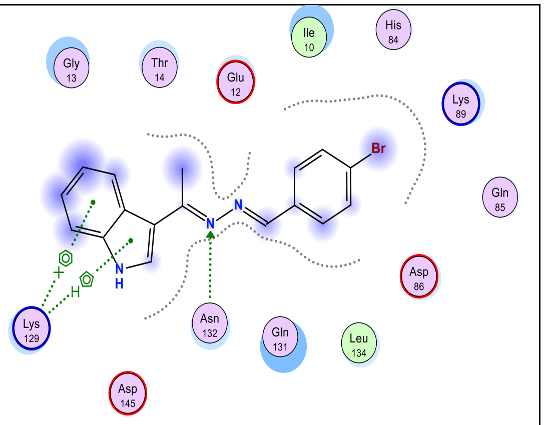
**3e**	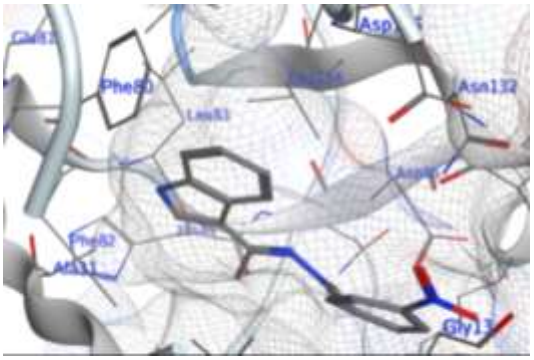	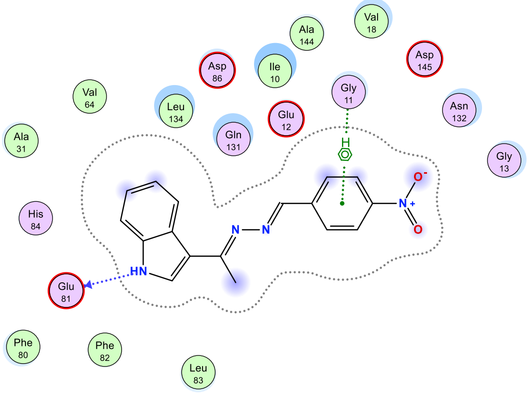
**3f**	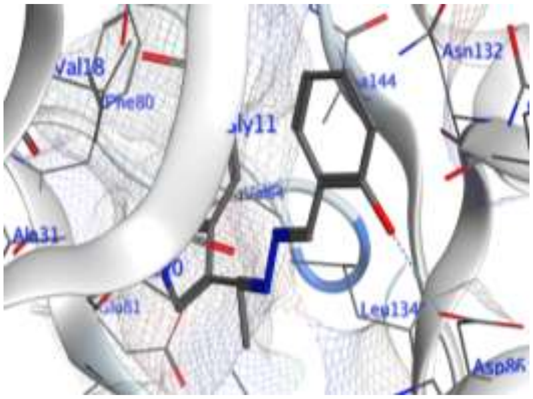	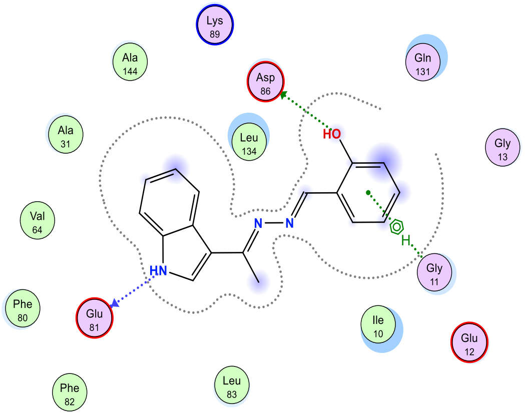

3g

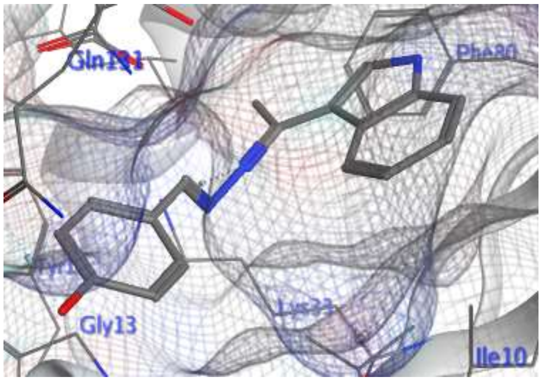
Gln131
Phe80
Gly13
Ile10

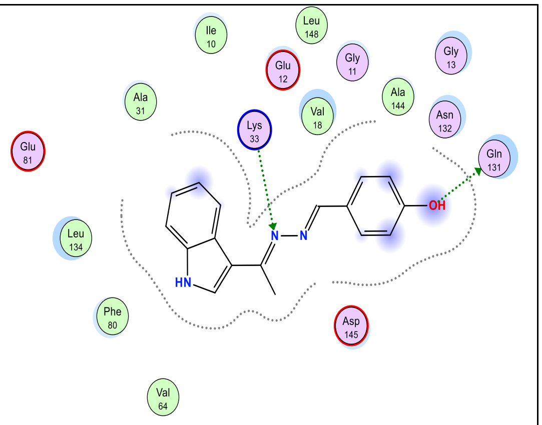
Ile 10
Leu 148
Glu 12
Gly 11
Gly 13
Ala 31
Lys 33
Val 18
Ala 144
Asn 132
Glu 81
Gln 131
OH
N
N
Leu 134
HN
Phe 80
Asp 145
Val 64

3h

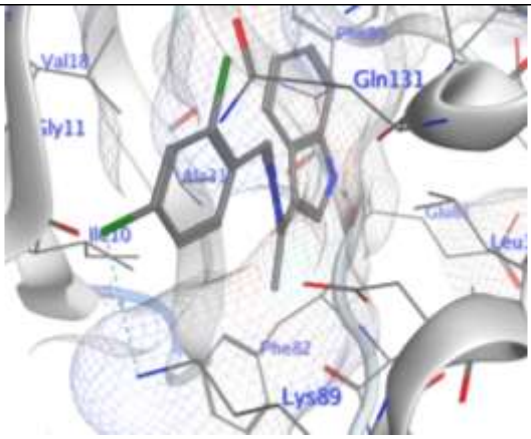
Val18
Gln131
Lys89

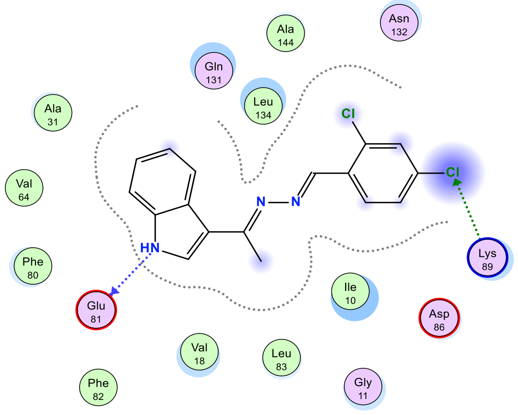
Ala 144
Asn 132
Gln 131
Ala 31
Leu 134
Cl
Val 64
N
N
Cl
Phe 80
HN
Lys 89
Ile 10
Glu 81
Asp 86
Val 18
Leu 83
Phe 82
Gly 11

7

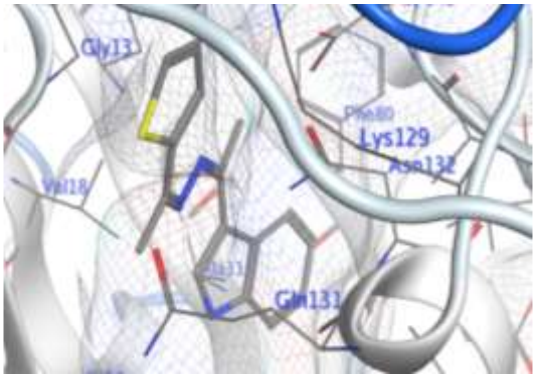
Gly13
Lys129
Asn132
Gln131

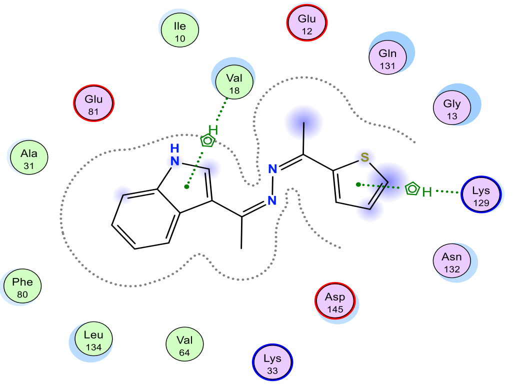
Ile 10
Glu 12
Gln 131
Val 18
Glu 81
Gly 13
H
Ala 31
H
N
S
N
N
H
Lys 129
Asn 132
Phe 80
Asp 145
Leu 134
Val 64
Lys 33

**9**	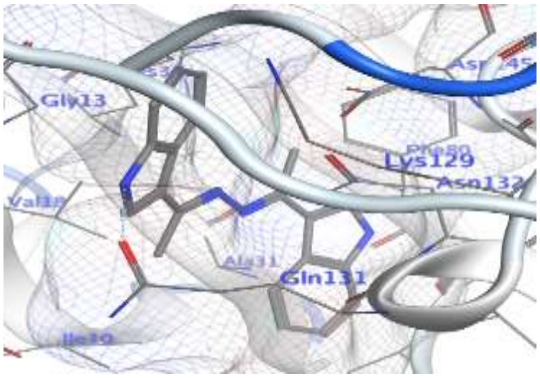	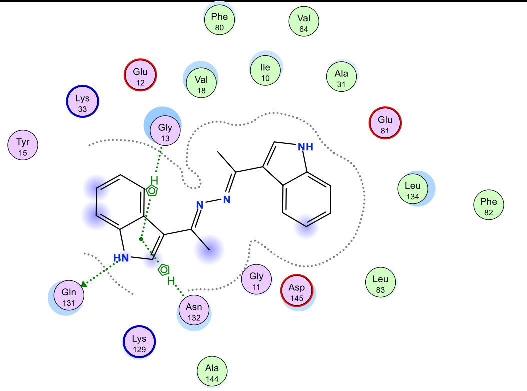
**11**	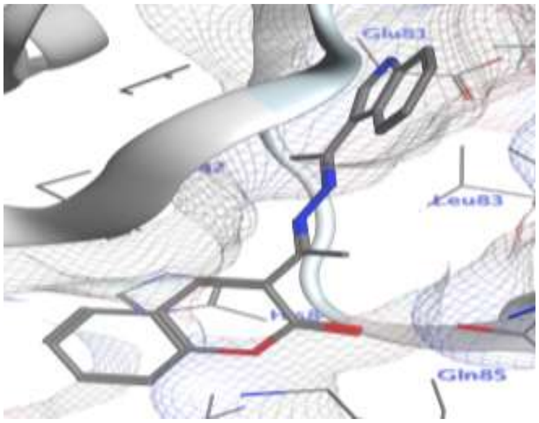	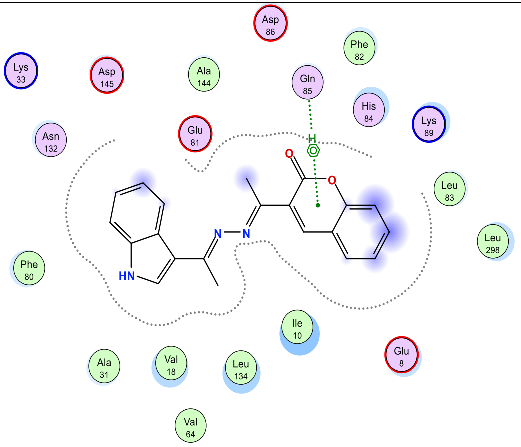
**13**	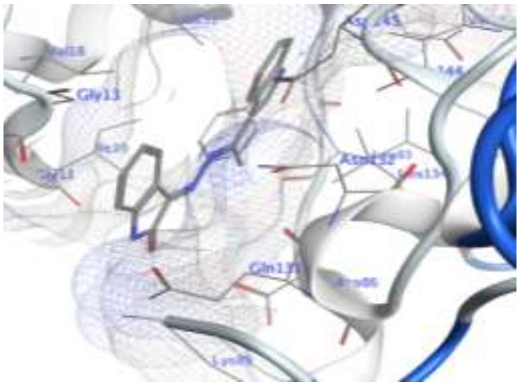	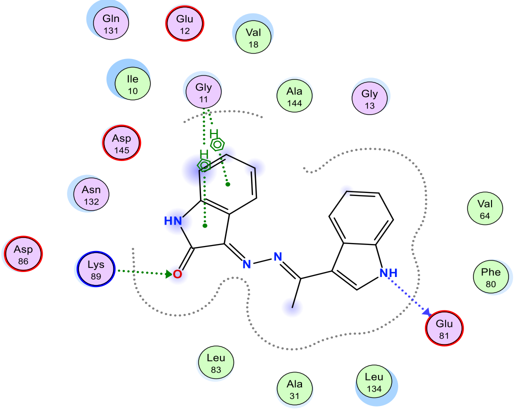

Figure S1. ligand interactions in 3D and 2D at the 3IG7 receptors' binding site for derivatives **3a-h, 7, 9, 11** and **13**.

Table S1. Computed values for prediction parameters of derivative **9**.

Parameter	Predicted values
**A. Physicochemical Properties**	
Formula	$C_{20}H_{19}N_4$
Number of heavy atoms	24
Number of aromatic heavy atoms	14
Fraction Csp3	0.15
Number of rotatable bonds	3
Number of *H*-bond acceptors	2
Number of *H*-bond donors	2
Molar Refractivity	101.41
TPSA (Total Polarity Surface Area)	56.30 Å²
Lipophilicity	+ve
Water Solubility	Moderately soluble
**B. Pharmacokinetics**	
Human intestine absorption (HIA)	High
Blood-brain barrier (BBB) permeation	Yes
P-glycoprotein substrate	Yes
Cytochrome P450 2C19, P450 2C9, P450 2D6, P450 3A4 inhibitors	No
Skin permeation	-4.25 cm/s
Drug- likeness	Yes; 0 violation
Bioavailability Score	0.55
**C. Medicinal Chemistry**	
Interference structures	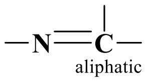
Lead-likeness	No
Synthetic accessibility: score from 1 (very easy) to 10 (very difficult)	3.74

Table S2. Computed values for prediction parameters of derivative **3d**.

Parameter	Predicted values
**A. Physicochemical Properties**	
Formula	$C_{17}H_{14}BrN_3$
Number of heavy atoms	21
Number of aromatic heavy atoms	15

Fraction Csp3	0.06
Number of rotatable bonds	3
Number of *H*-bond acceptors	2
Number of *H*-bond donors	1
Molar Refractivity	92.44
TPSA (Total Polarity Surface Area)	40.51 Å²
Lipophilicity	+ve
Water Solubility	Moderately soluble
**B. Pharmacokinetics**	
Human intestine absorption (HIA)	High
Blood-brain barrier (BBB) permeation	No
P-glycoprotein substrate	Yes
Cytochrome P450 2D6, P450 3A4 inhibitors	No
Skin permeation	-5.29 cm/s
Drug -likeness	Yes; 0 violation
Bioavailability Score	0.55
**C. Medicinal Chemistry**	
Interference structures	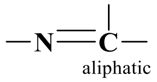
Lead-likeness	No
Synthetic accessibility: score from 1 (very easy) to 10 (very difficult)	2.33

Table S3. Computed values for prediction parameters of Doxorubicin.

Parameter	Predicted values
**A. Physicochemical Properties**	
Formula	$C_{27}H_{29}NO_{11}$
Number of heavy atoms	39
Number of aromatic heavy atoms	12
Fraction Csp3	0.44
Number of rotatable bonds	5
Number of *H*-bond acceptors	12
Number of *H*-bond donors	6
Molar Refractivity	132.66
TPSA (Total Polarity Surface Area)	206.07 Å²
Lipophilicity	+ve
Water Solubility	Moderately soluble

**B. Pharmacokinetics**	
Human intestine absorption (HIA)	Low
Blood-brain barrier (BBB) permeation	No
P-glycoprotein substrate	Yes
Cytochrome P450 2D6, P450 3A4 inhibitors	No
Skin permeation	-8.71 cm/s
Drug -likeness	No; 3 violations
Bioavailability Score	0.17
**C. Medicinal Chemistry**	
Interference structures	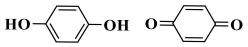
Lead-likeness	No
Synthetic accessibility: score from 1 (very easy) to 10 (very difficult)	5.81

3.2. Docking study

The molecular modeling for the synthesized derivatives was done using Chemdraw 12.0 and the molecular system design software version 2015.10. The scoring function, dock function (S, Kcal/mol) of the MOE software was used to calculate the ligands' binding affinities. The crystal structure of studied target was downloaded from Protein Data Bank (PDB ID: 3IG7), resolution: 1.80 Å was downloaded from the protein data bank in PDB format [48]. The enzyme was ready for investigations using docking: (i) The waters were eliminated from the protein [69]. (ii) Hydrogen atoms were added to the structure with their standard geometry [70] then reconnect the bonds broken and fixing the potential [71]**.** (iii) For the large site using MOE Alpha Site Finder, dummy atoms were added to the enzyme structure which generated from the resulting alpha spheres [72]. (iv) Examining how at the active site, the ligand interacts with the amino acids. We began by replacing the characterized ligand (EFP) with our manufactured ligands, and the diagrams in 2D and 3D were used to show how newly synthesized molecules interacted inside the active area of the CDK-5 enzyme (**Figure S1**). The most negative value for the active ligands is where the best docking score is found. According to established protocols, all docking operations and scoring were documented [57, 73]. Docking was done using the Triangle Matcher placement method and the London dG scoring tool. The overall docking results were compared with the native ligand's bound EFP crystal composition.
